# Sex-Based Heterogeneity in the Clinicopathological Characteristics and Prognosis of Breast Cancer: A Population-Based Analysis

**DOI:** 10.3389/fonc.2021.642450

**Published:** 2021-02-24

**Authors:** Yiqun Han, Jiayu Wang, Zijing Wang, Binghe Xu

**Affiliations:** Department of Medical Oncology, National Cancer Center/National Clinical Research Center for Cancer/Cancer Hospital, Chinese Academy of Medical Sciences and Peking Union Medical College, Beijing, China

**Keywords:** sex, male, breast cancer, clinicopathological features, prognosis

## Abstract

**Purpose:**

To better understand the differences in clinicopathological features and prognosis between male breast cancer (MBC) and female breast cancer (FBC).

**Material and Methods:**

Data on patients diagnosed with breast cancer from January 1, 2010, to December 31, 2016, were obtained from the Surveillance, Epidemiology, and End Results database. Selected patients were classified into MBC and FBC, of which population demographics and clinicopathological features at baseline were successively extracted for analysis. Comparative analysis was performed to explore the differences in baseline characteristics, followed by propensity-score matching to calibrate the objective distinctions for adjusted analysis. Survival analysis was carried out to investigate divergences presented in prognosis from the two cohorts, and risk factors for prognosis were successively identified using univariate and multivariate COX regression analyses.

**Results:**

A total of 407341 individuals were eligible, including 3111 MBC (0.7%) and 404230 FBC (99.3%) patients. Comparatively, patients with MBC tended to be older at diagnosis, with a higher confirmation of ductal carcinoma, a higher histological grade, a higher TNM stage, a higher proportion of luminal-like subtype, a higher rate of lung metastasis, a lower incidence of liver involvement, and a lower rate of surgical, radiation, and chemotherapeutic delivery. The overall prognosis of MBC was significantly worse than that of FBC, with a decreasing divergence both in median overall survival (65.5 months vs. 72.7 months, *P*<0.0001) and median breast cancer-specific survival (75.4 months vs. 77.8 months, *P*<0.0001). However, these discrepancies were not consistent among patients from different subgroups stratified by molecular subtype, age at diagnosis, or disease stage.

**Conclusion:**

In this study, sex-based heterogeneity in clinicopathological characteristics and prognostic profiles was observed in the overall population of patients with breast cancer and was significantly variable among different subgroups. A male-specific design with reasonable endpoints for a clinical trial protocol will be warranted in the future.

## Introduction

Breast cancer is a heterogeneous malignancy, with diverse inherent heterogeneities originating from comprehensive characteristics, and patient sex is a significant factor. Although male breast cancer (MBC) is rare, composing approximately 1% of the global breast cancer population, it has been occurring with an increasing incidence with an estimated 2300 newly diagnosed cases in 2017, 2500 cases in 2018, and 2670 cases in 2019 (1–3).

Historically, it has been acknowledged that MBC presents distinct profiles of clinicopathological features and prognostic outcomes in comparison with female breast cancer (FBC) (4–6). However, the majority of treatments for MBC are extrapolated from the standardized therapeutics of FBC (7), with limited consideration of the specific biological features as well as clinical presentations of male patients with breast cancer. It is essential to curate an informative understanding of this specific cohort in clinical practice.

Previous studies have evaluated the impact of patient sex on clinical features and prognostic profiles. However, the majority of studies were focused on a specified cohort, obtained findings from a limited sample size with cross-sectional performance, or did not adjust for potential bias from baseline characteristics (8–10). In addition, few studies have illustrated the correlation between sex-based heterogeneity and the presented characteristics and prognostic outcomes. Herein, we performed this analysis based on a large-scale population to comprehensively discuss the heterogeneous effects of sex on the clinical profiles of breast cancer, with the aim of creating a better understanding of the effects of sex and informative evidence for prospective practice.

## Methods

### Data Source and Cohort Selection

Population information was obtained from the Surveillance, Epidemiology, and End Results (SEER) database to create a cohort of patients diagnosed with breast cancer between January 1, 2010, and December 31, 2016 (November 2018 submission). Since human epidermal growth factor receptor 2 (HER2) status was registered beginning in 2010, this study adopted a cohort dataset for which the initial diagnosis occurred after 2010. The inclusion criteria were sex and clear survival outcomes. Patients with missing histopathological diagnoses and molecular subtypes were excluded.

In this study, patients were classified into MBC and FBC according to sex and clinicopathological characteristics, including age at diagnosis, race, primary site, cancer laterality, histologic type, grade, tumor size, nodal status, distant metastasis, molecular subtype, estrogen receptor (ER) status, progesterone receptor (PgR) status, HER2 status, surgical performance, radiation treatment, and chemotherapeutic delivery. Due to the publicly available nature of the data from the SEER database, this retrospective population-based study was exempted from approval by the ethics committee of the Chinese Academy of Medical Sciences. This study was conducted in accordance with the Strengthening the Reporting of Observational Studies in Epidemiology (STROBE) guidelines.

### Outcome Measures

For the current analysis, molecular subtypes of breast cancer were categorized into four classifications, including hormonal receptor (HR) positive/HER2 negative (HR+/HER2-), HR positive/HER2 positive (HR+/HER2+), HR negative/HER2 positive (HR-/HER2+, HER2 enriched), and HR negative/HER2 negative (HR-/HER2-, TN). Early breast cancer (eBC) was defined as a breast malignancy without distant metastasis detected by imaging techniques, while metastatic breast cancer (mBC) was defined as distant metastasis at the initial diagnosis. Young breast cancer referred to patients with a diagnosis of breast cancer at <40 years, while elderly breast cancer referred to those >70 years. Overall survival (OS) was defined as the interval between the initial diagnosis of breast cancer and death caused by any reason. Breast cancer-specific survival (BCSS) was defined as the period from the categorical diagnosis of breast cancer to death caused by cancer progression. According to SEER terminology, visceral metastases include liver, lung, and brain involvement. The American Joint Committee on Cancer 7^th^ edition guidelines were adopted to define the TNM staging of breast cancer.

### Statistical Analyses

The different profiles of population demographics and clinicopathological features between MBC and FBC were explored using Pearson’s chi-squared and Fisher’s exact probability tests for qualitative data and the t-test or Wilcoxon rank test for quantitative data with a normal and abnormal distribution, respectively. Propensity-score matching (PSM) was performed to calibrate objective distinctions between baseline characteristics of the two groups of breast cancer. The comparative analysis of prognosis was conducted using the Kaplan-Meier method with a log-rank test, in which subgroup analysis was stratified by molecular subtypes, disease stage, and diagnosed age. Risk factors of MBC with hazard ratios and 95% confidence intervals were investigated with successive univariate and multivariate analyses. All statistical analyses were two-sided with P values < 0.05 considered statistically significant and were performed using IBM SPSS Statistics, Version 26.0 (Armonk, NY, IBM Corp) and R software 3.6.4.

## Results

**Figure 1:**
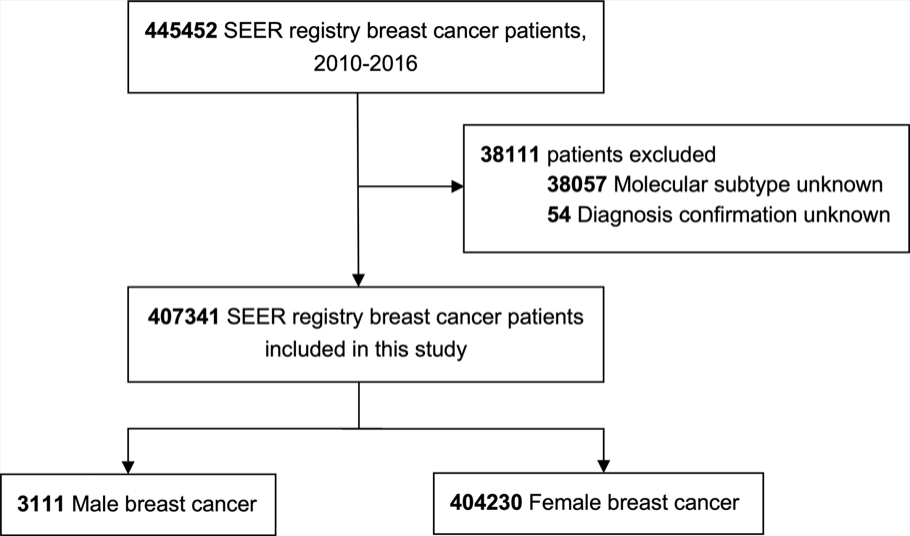
The flowchart of information processing and patient selection.

In this study, a total of 445452 patients were initially identified, of which 407341 individuals were eligible, including 3111 MBC patients (0.7%) and 404230 FBC patients (99.3%), **Figure 1** shows the cohort selection process. In the entire MBC population, 84.9% (2640/3111), listed were HR+/HER2-, 12.1% (377/3111) were HR+/HER2+, 0.9% (29/3111) were HR-/HER2+, and 2.1% (65/3111) were HR-/HER2-, while 92.0% (2863/3111) had early disease and 8.0% (248/3111) had metastatic cancer.

### Clinicopathological Characteristics

The results of comparative analyses indicated a substantial impact of sex on the disease profiles, of which, population demographics and baseline clinicopathological characteristics are listed in **Table 1**. The median ages of those with MBC and FBC were 67.11 years and 61.70 years, respectively. Compared to FBC, patients with MBC tended to be older at the time of breast cancer diagnosis (>60 years, 74.0% vs. 56.3%, *P*<0.0001) and had a higher occurrence of central localization (42.0% vs 4.7%, *P*<0.0001), an increased incidence of left disease (53.1% vs. 50.6%, *P*=0.016), a higher confirmation of ductal carcinoma (89.8% vs. 78.3%, *P*<0.0001), a higher histological grade (III-IV, 33.3% vs. 30.5%, *P*<0.0001), a larger tumor size (>5 cm, 12.1% vs. 10.2%, *P*<0.0001), a higher percentage of nodal involvement (N1-3, 37.6% vs. 27.0%, *P*<0.0001), an increasing existence of distant metastasis (M1, 8.0% vs. 5.2%, *P*<0.0001), a higher proportion of luminal-like subtype (97.0% vs. 84.2%, *P*<0.0001), and lower rates of undergoing surgery (89.5% vs. 91.1%, *P*=0.002), radiotherapy (28.1% vs. 48.8%, *P*=0.001), and chemotherapy (37.5% vs. 39.9%, *P*=0.006).

**Table 1:** Baseline characteristics of male breast cancer and female breast cancer.

Characteristics	Male (N=3111)		Female (N=404230)		*P* value
	N	Percent (%)	N	Percent (%)	
Age at diagnosis, median, y	67.11		61.70		<0.0001
Age group at diagnosis, y					<0.0001
<40	54	1.7	19122	4.7	
40-49	210	6.8	61240	15.1	
50-59	545	17.5	96134	23.8	
60-69	952	30.6	110731	27.4	
70-79	823	26.5	74900	18.5	
≥80	527	16.9	42103	10.4	
Race					<0.0001
White	2469	79.4	319544	79.1	
Black	463	14.9	44890	11.1	
Other	162	5.2	37024	9.2	
Unknown	17	0.5	2772	0.7	
Primary site					<0.0001
Upper-outer	360	11.6	135787	33.6	
Lower-outer	109	3.5	30259	7.5	
Upper-inner	128	4.1	49555	12.3	
Lower-inner	51	1.6	22488	5.6	
Central portion	1306	42.0	19116	4.7	
Nipple	158	5.1	1495	0.4	
Axillary tail	5	0.2	2194	0.5	
Overlapping	482	15.5	92179	22.8	
Unknown	512	16.5	51157	12.7	
Laterality					0.016
Right	1452	46.7	198932	49.2	
Left	1651	53.1	204360	50.6	
Bilateral	0	0.0	125	<0.01	
Unknown	8	0.3	813	0.2	
Histologic type					<0.0001
DC	2795	89.8	316491	78.3	
LC	95	3.1	63683	15.8	
Others	221	7.1	24056	6.0	
Grade					<0.0001
Grade 1	358	11.5	89848	22.2	
Grade 2	1566	50.3	172575	42.7	
Grade 3	1029	33.1	122391	30.3	
Grade 4	6	0.2	1033	0.3	
Unknown	152	4.9	18383	4.5	
T					<0.0001
T0/Tis	37	1.2	6056	1.5	
T1	1326	42.6	229366	56.7	
T2	1280	41.1	118153	29.2	
T3	102	3.3	24258	6.0	
T4	273	8.8	16841	4.2	
TX	93	3.0	9556	2.4	
N					<0.0001
N0/N1mi	1900	61.1	289330	71.6	
N1	752	24.2	75638	18.7	
N2	266	8.6	20352	5.0	
N3	151	4.9	12969	3.2	
NX	42	1.4	5941	1.5	
M					<0.0001
M0	2863	92.0	383345	94.8	
M1	248	8.0	20885	5.2	
Subtype					<0.0001
HR+/HER2-	2640	84.9	297068	73.5	
HR+/HER2+	377	12.1	43236	10.7	
HR-/HER2+	29	0.9	18526	4.6	
HR-/HER2-	65	2.1	45400	11.2	
ER					<0.0001
Positive	3008	96.7	335996	83.1	
Negative	102	3.3	68049	16.8	
Borderline	1	<0.01	152	<0.01	
Unknown	0	0.0	33	<0.01	
PgR					<0.0001
Positive	2787	89.6	291669	72.2	
Negative	312	10.0	111316	27.5	
Borderline	4	0.1	439	0.1	
Unknown	8	0.3	806	0.2	
HER2					0.001
Positive	406	13.1	61762	15.3	
Negative	2705	86.9	342468	84.7	
Surgery					0.002
Yes	2784	89.5	368132	91.1	
No/Unknown	327	10.5	36098	8.9	
Radiotherapy					0.001
Yes	873	28.1	197204	48.8	
No/Unknown	2238	71.9	207026	51.2	
Chemotherapy					0.006
Yes	1167	37.5	161457	39.9	
No/Unknown	1944	62.5	242773	60.1	

DC, ductal carcinoma; LC, lobular carcinoma; ER, estrogen receptor; PgR, progesterone receptor; HER2, human epidermal growth factor receptor 2.

Differences in molecular subtypes were consistently seen between MBC and FBC (**Supplementary Table 1**). The clinicopathological features and treatment options of HR+/HER2- MBC patients were consistent with those of the overall population and comparable to FBC patients, except for a few differences in the receipt of surgery (85.7% vs. 87.9%, *P*=0.186) and chemotherapy (68.2% vs. 70.7%, *P*=0.283). In contrast, there was no significant difference in age at diagnosis of HR-/HER2+ MBC or FBC, although the median age was 66.38 years for HR-/HER2- MBC and 59.21 years for HR-/HER2- FBC, indicating the advanced age trend in initial diagnosis among all subtypes. In comparison with FBC, HR-/HER2+ MBC tended to be lower in histologic grade (III-IV, 58.6% vs. 68.3%, *P*=0.008) and tumor size (>5 cm, 13.8% vs. 19.8%, *P*<0.0001), which is consistent with HR-/HER2- MBC (histologic grade, III-IV, 67.7% vs. 75.6%, *P*=0.008; tumor size, >5 cm, 7.7% vs. 15.3%, *P*<0.0001). The rate of surgical performance was significantly lower in both HR-/HER2+ (62.1% vs. 85.5%, *P*<0.0001) and HR-/HER2- MBC patients (64.6% vs. 89.5%, *P*<0.0001), while no significant differences were observed in the delivery of radiotherapy or chemotherapy.

Significant differences were observed in eBC and mBC between MBC and FBC (**Supplementary Tables 2 and 3**). In those with eBC, clinicopathological profiles and therapeutic options tended to be similar between MBC and FBC. However, there were no significant differences detected between MBC and FBC patients in disease laterality, tumor size, nodal metastasis, HER2 status, or treatment application in mBC.

For the mBC population, different patterns of metastases were seen in MBC and FBC. Overall, the rate of liver metastasis was significantly lower in MBC than in FBC (10.5% vs. 24.5%, *P*<0.0001), while the incidence of lung involvement was comparatively higher in MBC (37.5% vs. 29.8%, *P*=0.033). No apparent difference was detected in the proportion of bone and brain metastases between the two groups (**Supplementary Table 3**). The rates of liver-only disease (1.2% vs. 6.5%, *P*<0.0001) and paired metastases involving bone and liver (2.8% vs. 7.6%, *P*=0.005) were relatively lower in MBC. In contrast, paired metastases in bone and lung (17.3% vs. 9.1%, *P*<0.0001), in addition to synchronous metastases in bone, lung, and brain (3.6% vs. 1.0%, *P*=0.006), were significantly higher in MBC than in FBC (**Supplementary Table 4**).

### Survival Outcomes

**Figure 2:**
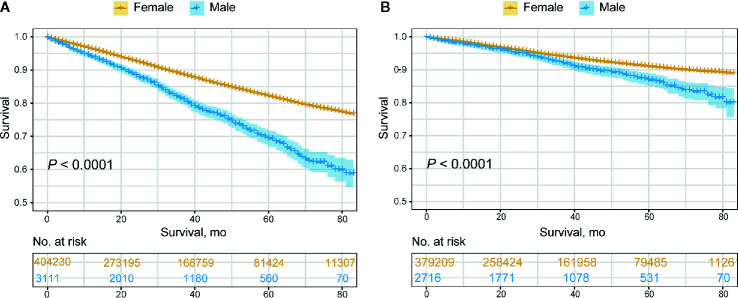
Overall prognosis of male breast cancer and female breast cancer. **(A)** Overall survival. **(B)** Breast cancer-specific survival.

**Figure 3:**
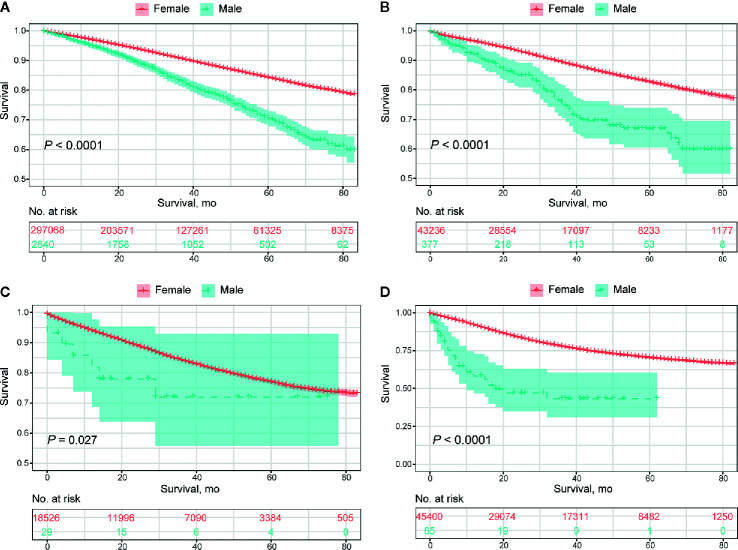
Overall prognosis of male breast cancer and female breast cancer concerning molecular subtypes. **(A)** HR+/HER2- subtype. **(B)** HR+/HER2+ subtype. **(C)** HER2 enriched subtype. **(D)** Triple-negative subtype.

Collectively, the overall prognosis of MBC was significantly worse than that of FBC, with a decreasing divergence both in overall survival (median OS, 65.5 months vs. 72.7 months, *P*<0.0001) and BCSS (median BCSS, 75.4 months vs. 77.8 months, *P*<0.0001) (**Figure 2**). This tendency remained consistent in those classified as HR+/HER2-, HR+/HER2+, and HR-/HER2-, while no significant difference was detected in the HR-/HER2+ subgroup (**Figure 3**; **Supplementary Figure 1**). The OS of elderly MBC was significantly worse than that of elderly FBC (median OS, 60.0 months vs. 65.3 months, *P*<0.0001), whereas there were no significant differences in the OS of younger patients or in BCSS from the paired groups (**Supplementary Figure 2 and Table 5**). Regarding therapeutic options, both OS and BCSS were consistently worse in MBC compared to FBC with the receipt of surgery, radiotherapy, and chemotherapy (**Supplementary Figure 3**). In addition, no significant difference was observed in patients with organ-specific involvement in MBC or FBC (**Supplementary Figure 4**).

To eliminate the objective baseline differences between the two groups, we performed PSM in a 1:2 ratio to further quantitatively investigate the impact of sex on the prognostic profiles. Overall, male patients exhibited a relatively worse OS than female patients with breast cancer (median OS, 65.6 months vs. 68.3 months, *P*<0.0001), while there was no significant difference in BCSS between the two groups (**Supplementary Figure 5**). The prognosis of the HR-/HER2- subgroup was worse in MBC than in FBC, with a significantly shortened OS (median OS, 32.1 months vs. 63.3 months, *P*<0.0001) and BCSS (median OS, 36.6 months vs. 71.2 months, *P*<0.0001), whereas no comparative distinctions were detected in HR+/HER2-, HR+/HER2+, and HR-/HER2+ subgroups (**Supplementary Figure 6 and Figure 7**). There were no significant differences in the prognosis of both young and elderly patients with MBC and FBC (**Supplementary Figure 8 and Table 6**). Furthermore, the OS was consistently inferior in MBC with the performance of surgery, radiotherapy, and chemotherapy, while no statistical significance was detected in BCSS associated with surgical and chemotherapeutic intervention between MBC and FBC (**Supplementary Figure 9**).

For male patients with breast cancer, survival outcomes were successively evaluated with stratification by clinicopathological characteristics (**Supplementary Table 7**). OS tended to decrease with age, while BCSS was weakly associated with the age of diagnosis. For MBC, patients of African descent had the worst prognosis, and the histological type of lobular carcinoma was favorable for survival. Both OS and BCSS were successively shortened with a histological grade as well as TNM stage of tumor size, nodal status, and distant metastasis, and patients with HR-/HER2- disease demonstrated relatively worse OS and BCSS in comparison with the other three subtypes. Patients who underwent surgery and chemotherapy showed a significantly improved prognosis, whereas radiation treatment did not influence the survival outcomes of MBC. This kind of association between clinicopathological variables and prognostic profiles was consistent with findings from univariate analysis, which were further investigated by multivariate analysis (**Table 2**; **Supplementary Table 8**). Age at diagnosis, race, tumor size, nodal status, distant metastasis, molecular subtype, surgical implementation, chemotherapeutic delivery, and liver and brain involvement had independent effects on OS, and all but the age at diagnosis and visceral involvement were significantly related to BCSS.

**Table 2:** Univariate and multivariate COX regression analyses of prognostic factors for overall survival in male breast cancer.

Characteristics	Univariate			Multivariate		
	Hazard ratio	95%CI	*P* value	Hazard ratio	95%CI	*P* value
Age group at diagnosis, y			<0.0001			<0.0001
<40	Reference			Reference		
40-49	1.33	0.51-3.42	0.555	1.71	0.65-4.50	0.274
50-59	1.33	0.54-3.29	0.537	1.73	0.69-4.34	0.246
60-69	1.48	0.61-3.61	0.387	2.13	0.86-5.29	0.103
70-79	1.96	0.80-4.77	0.139	3.08	1.24-7.67	0.015
≥80	4.20	1.73-10.20	0.002	5.92	2.37-14.82	<0.0001
Race			0.002			0.005
White	Reference			Reference		
Black	1.38	1.13-1.69	0.002	1.34	1.08-1.66	0.008
Others	0.77	0.50-1.18	0.228	0.71	0.46-1.09	0.119
Primary Site			<0.0001			0.434
Upper-outer	Reference			Reference		
Lower-outer	0.78	0.44-1.38	0.395	0.89	0.50-1.60	0.699
Upper-inner	1.08	0.64-1.82	0.781	1.24	0.72-2.12	0.431
Lower-inner	1.30	0.66-2.55	0.450	1.30	0.65-2.59	0.452
Central	1.24	0.92-1.67	0.158	1.07	0.79-1.45	0.680
Overlapping	1.09	0.77-1.55	0.624	0.98	0.68-1.39	0.896
Others	1.36	0.89-2.08	0.151	1.21	0.79-1.87	0.387
Unknown	2.06	1.51-2.82	<0.0001	1.31	0.94-1.82	0.109
Laterality			0.065			/
Right	Reference			/		
Left	1.01	0.86-1.19	0.892	/	/	/
Others	3.24	1.21-8.69	0.020	/	/	/
Histologic type			0.007			0.479
DC	Reference			Reference		
LC	0.78	0.45-1.36	0.388	0.71	0.40-1.24	0.231
Others	1.50	1.15-1.95	0.003	1.02	0.74-1.41	0.886
Grade			<0.0001			0.002
Grade 1	Reference			Reference		
Grade 2	1.26	0.93-1.72	1.150	1.00	0.73-1.37	0.996
Grade 3	2.00	1.47-2.73	<0.0001	1.45	1.05-2.01	0.023
Grade 4	6.60	2.38-18.34	<0.0001	1.27	0.39-4.17	0.695
Unknown	3.63	2.47-5.33	<0.0001	1.35	0.88-2.07	0.174
T			<0.0001			<0.0001
T0/Tis/T1	Reference			Reference		
T2	1.88	1.55-2.28	<0.0001	1.61	1.32-1.96	<0.0001
T3	3.49	2.40-5.06	<0.0001	2.48	1.67-3.66	<0.0001
T4	4.16	3.28-5.28	<0.0001	2.08	1.56-2.77	<0.0001
TX	3.54	2.45-5.12	<0.0001	0.98	0.61-1.56	0.927
N			<0.0001			0.012
N0/N1mi	Reference			Reference		
N1	1.63	1.35-1.96	<0.0001	1.21	0.98-1.50	0.073
N2	1.75	1.35-2.27	<0.0001	1.44	1.07-1.92	0.015
N3	1.70	1.22-2.38	<0.0001	1.25	0.86-1.80	0.238
NX	5.93	3.77-9.34	<0.0001	2.49	1.32-4.68	0.005
M			<0.0001			0.002
M0	Reference			Reference		
M1	5.50	4.53-6.67	<0.0001	1.98	1.29-3.04	0.002
Subtype			<0.0001			<0.0001
HR+/HER2-	Reference			Reference		
HR+/HER2+	1.32	1.04-1.68	0.020	1.27	0.99-1.64	0.061
HR-/HER2+	1.84	0.91-3.69	0.089	1.02	0.47-2.24	0.952
HR-/HER2-	5.53	3.82-8.02	<0.0001	4.76	3.10-7.30	<0.0001
Surgery			<0.0001			<0.0001
Yes	Reference			Reference		
No/Unknown	5.87	4.88-7.06	<0.0001	2.55	1.95-3.33	<0.0001
Radiotherapy			0.472			/
Yes	Reference			/		
No/Unknown	1.07	0.89-1.28	0.472	/	/	/
Chemotherapy			0.004			0.003
Yes	Reference			Reference		
No/Unknown	5.94	4.63-7.61	0.004	1.39	1.12-1.72	
Visceral metastasis			<0.0001			0.803
No	Reference			Reference		
Yes	0.44	0.26-0.75	<0.0001	1.31	0.58-2.97	0.513
Unknown	2.28	1.34-3.87	0.002	0.94	0.11-8.27	0.958
Bone			<0.0001			0.130
No	Reference			Reference		
Yes	5.79	4.67-7.20	<0.0001	3.01	0.60-15.15	0.182
Unknown	2.20	1.27-3.82	0.005	4.04	0.79-20.70	0.094
Liver			<0.0001			0.018
No	Reference			Reference		
Yes	7.98	4.98-12.78	<0.0001	22.19	1.57-314.50	0.022
Unknown	2.33	1.42-3.83	0.001	1.79	0.92-3.51	0.089
Lung			<0.0001			0.313
No	Reference			Reference		
Yes	5.36	4.09-7.04	<0.0001	2.21	0.67-7.28	0.193
Unknown	2.62	1.64-4.20	<0.0001	1.60	0.41-6.22	0.498
Brain			<0.0001			0.007
No	Reference			Reference		
Yes	14.79	8.64-25.32	<0.0001	12.51	0.86-180.99	0.064
Unknown	2.26	1.38-3.72	0.001	4.68	0.34-64.08	0.248

DC, ductal carcinoma; LC, lobular carcinoma; ER, estrogen receptor; PgR, progesterone receptor; HER2, human epidermal growth factor receptor 2.

## Discussion

To our knowledge, this is one of few studies to explore the effects of sex in breast cancer by undertaking a large-scale cohort analysis, and the first study to focus on diverse presentations systematically stratified into specific subgroups. Comparatively, male patients tended to be older at diagnosis, with more aggressive disease characteristics, and a relatively worse prognosis compared to the whole population, while substantial differences were detected across different subgroups.

In this study, the overall proportion of MBC accounted for 0.7% of the whole breast cancer population, which was in line with the incidence reported by William et al., and within the approximate range of 0.6-1%, as summarized in previous studies (11, 12). Luminal-like MBC was most common (97%) and the HER2-enriched MBC subtype was the rarest (0.9%), which was in accordance with previously reported proportions (13). To elaborate on the distinctions in clinicopathological characteristics, we performed a comparative analysis of those with MBC and FBC. Overall, male patients with breast cancer tended to be approximately 6 years older at diagnosis compared to females, which was consistent with previous findings (67 years vs. 62 years) (14) and had more aggressive cancer characteristics than female patients, primarily including a higher histologic grade, a significantly higher TNM stage, and a greater proportion of luminal-like disease, which was in accordance with previous findings (15, 16). There was no significant difference in age at diagnosis in the HER2-enriched population, and no race-related difference was detected between HER2-enriched and TN subtypes in MBC or FBC. Moreover, the proportions of ductal carcinoma, histologic grade, and tumor size were consistently lower in MBC than in FBC for these two subtypes. Given the substantial proportion of initially advanced diseases, we also focused on differences in metastatic patterns between the sexes. The rates of liver involvement both in a single presentation and in combination with bone metastasis were lower, while the overall incidence of lung metastasis was significantly higher in MBC than in FBC. However, these differences did not affect the organ-specific prognosis of the two groups.

The overall prognosis of MBC was relatively worse in terms of both OS and BCSS than that of FBC, except that the difference in BCSS was not significant after PSM analysis, indicating similar cancer-related mortality in the two groups. The overall prognosis of luminal-like MBC was consistently worse than that in FBC, while no significance was detected in BCSS after adjustment for baseline characteristics. The prognosis of TN MBC patients was generally worse than that of FBC patients, but this was not seen in HER2-enriched disease between MBC and FBC. Previous studies have assessed the comparative prognosis of MBC and FBC, which have generally seen worse prognoses in MBC (11, 12, 17). However, one potential hypothesis leading to this outcome could be due to more advanced age at diagnosis, with a diminishing life expectancy. To evaluate this discrepancy associated with the onset pattern, we performed a comparative adjusted survival analysis of young and elderly patients with MBC and FBC, which revealed that there were limited age-specific differences in the prognoses of patients with MBC and FBC and that the well-confirmed age tendency at diagnosis could account for this seemingly divergent prognosis between the two groups. Considering the great impact of therapeutic options on survival outcomes, we evaluated the prognostic heterogeneity between MBC and FBC patients relevant to the receipt of multiple treatments. The overall prognosis of both OS and BCSS was consistently inferior in MBC when compared to FBC, while this kind of difference was eliminated in BCSS regarding surgical intervention and chemotherapeutic delivery after the adjusted matching. No statistical significance was detected in the comparative prognosis between MBC and FBC associated with specific organic involvement.

In summary, the overall survival of MBC was significantly worse than that of FBC. There are several potential reasons for this phenomenon. First, our results suggested that disease characteristics tended to be more aggressive in males, yet the therapeutic rate was relatively lower, which worsened the survival outcomes of MBC patients. In addition to the limited degree of surgery and chemotherapy, although over 90% of MBC patients were HR-positive, a lower receipt of endocrine therapy was clearly reported (18). Male patients also tend to be excluded from clinical trial protocols, in which the male enrollment rate is 0.087% and the total proportion of MBC patients is 0.95%, as reported by Corrigan and colleagues (19). This severe imbalance could lead to the absence of sufficient data of efficacy and safety profiles from clinical trials, resulting in a limited treatment paradigms established for this group of patients (20). Of note, survival outcomes could vary significantly from different perspectives, such as disease subtypes and reason-specific death. Accordingly, more caution should be exercised regarding subject enrollment and endpoint establishment for both clinical trials and real-world studies.

In addition, our findings revealed that MBC is an age-associated malignancy with a steadily increasing risk of occurrence, which could be the result of the absence of periodical hormone fluctuations, unlike females. Advanced age at diagnosis, African descent, higher histologic grade, advanced TNM staging, TN subtype, and limited accessibility to surgery and chemotherapy were significant risk factors for OS of MBC, which were also broadly applicable to BCSS, while no significance was revealed for survival benefits from radiation performance. This study quantitatively investigated survival outcomes stratified by clinical features and curated a comprehensive body of risk factors for both OS and BCSS, which could achieve a better understanding of sex-based characteristics and provide strong evidence for the management of MBC.

Recent studies have postulated that the genetic characteristics of male patients are inherently distinct from those of female patients, especially regarding the sex-based disparity in immunologic reactions, in which females tend to respond more actively to immune-related stimulations and have a tendency for immunoediting (21, 22). This genetic heterogeneity should be considered, especially given that cancer immunotherapy has been rapidly evolving, and evidence has emerged that the corresponding efficacy of immune checkpoint inhibitors could vary by sex (23, 24). In addition, genetic profiles in the predisposition to cancer ontology are important, including the undetermined correlation between familial breast cancer and *BRCA* mutations in MBC, a different frequency of mutations in *BRCA1* and *BRCA2*, and discordant contribution of genetic variation to the cancer susceptibility between MBC and FBC (25–27). From these perspectives, both multidisciplinary therapeutic protocols and clinical trial designs should consider sex to further eliminate bias and curate an increasingly precise benefit for patients.

In conclusion, this study comprehensively assessed sex-based differences in clinicopathological features and survival outcomes between MBC and FBC. Male patients with breast cancer presented profoundly heterogeneous profiles that were distinct from those of FBC, which could vary distinctly with stratification by molecular subtypes, age at diagnosis, and disease stage. Considering the substantial differences associated with sex, current therapeutic options for females may not be appropriate for males. Clinical trials raising sufficient caution that are open to male patients in addition to well-performed studies based on real-world practice are warranted in the future.

### Limitations

This study has several limitations. First, therapeutic information regarding endocrine therapy is not available in this database. Accordingly, we could not investigate the potential correlations between endocrine therapy and prognosis, despite the leading proportion of endocrine-related MBC and potentially improved survival due to endocrine treatment (28). Second, selection bias could not be fully avoided, considering the extensive period of patient adoption over recent decades, covering approximately 40 years with the involvement of 34.6% of the United States population. Third, the sites involved after disease progression are not fully recorded in the SEER database, as data on local recurrence or distant sites are unavailable, which could lead to misestimation of survival outcomes associated with metastatic patterns. Lastly, some significant characteristics of breast cancer were absent in this database, including the Ki67 index, lymphovascular invasion, and pathologic features confirmed by recent studies such as tumor-infiltrating lymphocytes and fibrotic focus (29).

## Data Availability Statement

The original contributions presented in the study are included in the article/**Supplementary Material**. Further inquiries can be directed to the corresponding authors.

## Ethics Statement

Ethical review and approval was not required for the study on human participants in accordance with the local legislation and institutional requirements. Written informed consent for participation was not required for this study in accordance with the national legislation and the institutional requirements.

## Author Contributions

Conception and design: BX, JW, and YH. Development of methodology: YH. Acquisition of data: YH and ZW. Analysis and interpretation of data: YH, BX, JW, and ZW. Writing of the manuscript: YH. Review and revision of the manuscript: BX, JW, and ZW. Study supervision: BX and JW. All authors contributed to the article and approved the submitted version.

## Conflict of Interest

The authors declare that the research was conducted in the absence of any commercial or financial relationships that could be construed as a potential conflict of interest.
