# Supplementary material for: Sex-Based Heterogeneity in the Clinicopathological Characteristics and Prognosis of Breast Cancer: A Population-Based Analysis
Source: Front Oncol. 2021 Feb 24;11:642450. doi: 10.3389/fonc.2021.642450 (PMC7945032; doi:10.3389/fonc.2021.642450)
Supplement: Supplementary file 1 [file DataSheet_1.docx]

**Supplementary Materials**

Supplementary Table1. Baseline characteristics in MBC and FBC stratified by molecular subtypes.

Supplementary 2. Baseline characteristics of early disease from MBC and FBC.

Supplementary Table3. Baseline characteristics of metastatic disease from MBC and FBC.

Supplementary Table4. Metastatic patterns in MBC and FBC.

Supplementary Table5. Comparative OS and BCSS of MBC and FBC.

Supplementary Table6. Comparative OS and BCSS of MBC and FBC.

Supplementary Table7. The OS and BCSS of MBC stratified by clinicopathological characteristics.

Supplementary Table8. Risk factors for BCSS of MBC.

Supplementary Figure1. BCSS of MBC and FBC associated with molecular subtypes.

Supplementary Figure2. Overall prognosis of MBC and FBC regarding age.

Supplementary Figure3. Overall prognosis of MBC and FBC regarding therapeutics.

Supplementary Figure4. Overall prognosis of MBC and FBC regarding organ-specific involvement.

Supplementary Figure5. Overall prognosis of MBC and FBC associated with molecular subtypes after a 1:2 PSM.

Supplementary Figure6. OS of MBC and FBC associated with molecular subtypes after a 1:2 PSM.

Supplementary Figure7. BCSS of MBC and FBC associated with molecular subtypes after a 1:2 PSM.

Supplementary Figure8. Overall prognosis of MBC and FBC regarding age after a 1:2 PSM.

Supplementary Figure9. Overall prognosis of MBC and FBC regarding therapeutics after a 1:2 PSM.

**Supplementary Table1. Baseline characteristics in MBC and FBC stratified by molecular subtypes.**

| **Characteristics** | **HR+/HER2-** | | | | | ***P* value** | | **HR+/HER2+** | | | | | | ***P* value** | | **HR-/HER2+** | | | | | | ***P* value** | | **HR-/HER2-** | | | | | | | ***P* value** |  |
| --- | --- | --- | --- | --- | --- | --- | --- | --- | --- | --- | --- | --- | --- | --- | --- | --- | --- | --- | --- | --- | --- | --- | --- | --- | --- | --- | --- | --- | --- | --- | --- | --- |
|  | **MBC (N=2640)** | | **FBC (N=297068)** | | |  |  | **MBC (N=377)** | | | **FBC (N=43236)** | | |  |  | **MBC (N=29)** | | | **FBC (N=18526)** | | |  |  | **MBC (N=65)** | | | | **FBC (N=45400)** | | |  |  |
|  | N | Percent (%) | | N | Percent (%) | |  | | N | Percent (%) | | N | Percent (%) | |  | | N | Percent (%) | | N | Percent (%) | |  | | N | Percent (%) | | | N | Percent (%) |  | |
| Age at diagnosis, median, y | 67.67 |  | | 62.78 |  | | <0.0001 | | 63.83 |  | | 58.29 |  | | <0.0001 | | 61.03 |  | | 58.42 |  | | 0.295 | | 66.38 | |  | | 59.21 |  | <0.0001 | |
| Age group at diagnosis, y |  |  | |  |  | | <0.0001 | |  |  | |  |  | | <0.0001 | |  |  | |  |  | | 0.539 | |  |  | | |  |  | 0.004 | |
| <40 | 44 | 1.7 | | 10230 | 3.4 | |  | | 9 | 2.4 | | 3648 | 8.4 | |  | | 1 | 3.4 | | 1464 | 7.9 | |  | | 0 | 0.0 | | | 3780 | 8.3 |  | |
| 40-49 | 162 | 6.1 | | 41850 | 14.1 | |  | | 36 | 9.5 | | 8258 | 19.1 | |  | | 6 | 20.7 | | 3187 | 17.2 | |  | | 6 | 9.2 | | | 7945 | 17.5 |  | |
| 50-59 | 445 | 16.9 | | 67147 | 22.6 | |  | | 83 | 22.0 | | 11757 | 27.2 | |  | | 5 | 17.2 | | 5590 | 30.2 | |  | | 12 | 18.5 | | | 11640 | 25.6 |  | |
| 60-69 | 789 | 29.9 | | 84553 | 28.5 | |  | | 128 | 34.0 | | 10476 | 24.2 | |  | | 10 | 34.5 | | 4532 | 24.5 | |  | | 25 | 38.5 | | | 11170 | 24.6 |  | |
| 70-79 | 721 | 27.3 | | 59786 | 20.1 | |  | | 84 | 22.3 | | 5815 | 13.4 | |  | | 5 | 17.2 | | 2454 | 13.2 | |  | | 13 | 20.0 | | | 6845 | 15.1 |  | |
| ≥80 | 479 | 18.1 | | 33502 | 11.3 | |  | | 37 | 9.8 | | 3282 | 7.6 | |  | | 2 | 6.9 | | 1299 | 7.0 | |  | | 9 | 13.8 | | | 4020 | 8.9 |  | |
| Race |  |  | |  |  | | <0.0001 | |  |  | |  |  | | 0.001 | |  |  | |  |  | | 0.483 | |  |  | | |  |  | 0.528 | |
| White | 2108 | 79.8 | | 240644 | 81.0 | |  | | 300 | 79.6 | | 33151 | 76.7 | |  | | 19 | 65.5 | | 13346 | 72.0 | |  | | 42 | 64.6 | | | 32403 | 71.4 |  | |
| Black | 382 | 14.5 | | 27754 | 9.3 | |  | | 58 | 15.4 | | 5155 | 11.9 | |  | | 7 | 24.1 | | 2659 | 14.4 | |  | | 16 | 24.6 | | | 9322 | 20.5 |  | |
| Other | 134 | 5.1 | | 26611 | 9.0 | |  | | 18 | 4.8 | | 4612 | 10.7 | |  | | 3 | 10.3 | | 2384 | 12.9 | |  | | 7 | 10.8 | | | 3417 | 7.5 |  | |
| Unknown | 16 | 0.6 | | 2059 | 0.7 | |  | | 1 | 0.3 | | 318 | 0.7 | |  | | 0 | 0.0 | | 137 | 0.7 | |  | | 0 | 0.0 | | | 258 | 0.6 |  | |
| Primary site |  |  | |  |  | | <0.0001 | |  |  | |  |  | | <0.0001 | |  |  | |  |  | | <0.0001 | |  |  | | |  |  | <0.0001 | |
| Upper-outer | 303 | 11.5 | | 99282 | 33.4 | |  | | 39 | 10.3 | | 13929 | 32.2 | |  | | 5 | 17.2 | | 5920 | 32.0 | |  | | 13 | 20.0 | | | 16656 | 36.7 |  | |
| Lower-outer | 91 | 3.4 | | 22335 | 7.5 | |  | | 13 | 3.4 | | 3445 | 8.0 | |  | | 2 | 6.9 | | 1369 | 7.4 | |  | | 3 | 4.6 | | | 3110 | 6.9 |  | |
| Upper-inner | 112 | 4.2 | | 37288 | 12.6 | |  | | 16 | 4.2 | | 4705 | 10.9 | |  | | 0 | 0.0 | | 1806 | 9.7 | |  | | 0 | 0.0 | | | 5756 | 12.7 |  | |
| Lower-inner | 43 | 1.6 | | 16561 | 5.6 | |  | | 5 | 1.3 | | 2338 | 5.4 | |  | | 0 | 0.0 | | 969 | 5.2 | |  | | 3 | 4.6 | | | 2620 | 5.8 |  | |
| Central portion | 1130 | 42.8 | | 14440 | 4.9 | |  | | 164 | 43.5 | | 2227 | 5.2 | |  | | 5 | 17.2 | | 911 | 4.9 | |  | | 7 | 10.8 | | | 1538 | 3.4 |  | |
| Nipple | 143 | 5.4 | | 1056 | 0.4 | |  | | 14 | 3.7 | | 190 | 0.4 | |  | | 0 | 0.0 | | 139 | 0.8 | |  | | 1 | 1.5 | | | 110 | 0.2 |  | |
| Axillary tail | 3 | 0.1 | | 1522 | 0.5 | |  | | 0 | 0.0 | | 244 | 0.6 | |  | | 2 | 6.9 | | 78 | 0.4 | |  | | 0 | 0.0 | | | 350 | 0.8 |  | |
| Overlapping | 397 | 15.0 | | 68518 | 23.1 | |  | | 60 | 15.9 | | 9708 | 22.5 | |  | | 5 | 17.2 | | 4089 | 22.1 | |  | | 20 | 30.8 | | | 9864 | 21.7 |  | |
| Unknown | 418 | 15.8 | | 36066 | 12.1 | |  | | 66 | 17.5 | | 6450 | 14.9 | |  | | 10 | 34.5 | | 3245 | 17.5 | |  | | 18 | 27.7 | | | 5396 | 11.9 |  | |
| Laterality |  |  | |  |  | | 0.670 | |  |  | |  |  | | 0.713 | |  |  | |  |  | | 0.704 | |  |  | | |  |  | <0.0001 | |
| Right | 1240 | 47.0 | | 146883 | 49.4 | |  | | 173 | 45.9 | | 21045 | 48.7 | |  | | 11 | 37.9 | | 8971 | 48.4 | |  | | 28 | 43.1 | | | 22033 | 48.5 |  | |
| Left | 1395 | 52.8 | | 149548 | 50.3 | |  | | 203 | 53.8 | | 22078 | 51.1 | |  | | 18 | 62.1 | | 9497 | 51.3 | |  | | 35 | 53.8 | | | 23237 | 51.2 |  | |
| Bilateral | 0 | 0.0 | | 77 | <0.01 | |  | | 0 | 0.0 | | 19 | <0.01 | |  | | 0 | 0.0 | | 13 | 0.1 | |  | | 0 | 0.0 | | | 16 | <0.01 |  | |
| Unknown | 5 | 0.2 | | 560 | 0.2 | |  | | 1 | 0.3 | | 94 | 0.2 | |  | | 0 | 0.0 | | 45 | 0.2 | |  | | 2 | 3.1 | | | 114 | 0.3 |  | |
| Histologic type |  |  | |  |  | | <0.0001 | |  |  | |  |  | | <0.0001 | |  |  | |  |  | | <0.0001 | |  |  | | |  |  | <0.0001 | |
| DC | 2385 | 90.3 | | 222692 | 75.0 | |  | | 349 | 92.6 | | 37679 | 87.1 | |  | | 19 | 65.5 | | 16706 | 90.2 | |  | | 42 | 64.6 | | | 39414 | 86.8 |  | |
| LC | 85 | 3.2 | | 58086 | 19.6 | |  | | 8 | 2.1 | | 3712 | 8.6 | |  | | 0 | 0.0 | | 508 | 2.7 | |  | | 2 | 3.1 | | | 1377 | 3.0 |  | |
| Others | 170 | 6.4 | | 16290 | 5.5 | |  | | 20 | 5.3 | | 1845 | 4.3 | |  | | 10 | 34.5 | | 1312 | 7.1 | |  | | 21 | 32.3 | | | 4609 | 10.2 |  | |
| Grade |  |  | |  |  | | <0.0001 | |  |  | |  |  | | 0.011 | |  |  | |  |  | | 0.008 | |  |  | | |  |  | 0.003 | |
| Grade 1 | 346 | 13.1 | | 85890 | 28.9 | |  | | 8 | 2.1 | | 2741 | 6.3 | |  | | 2 | 6.9 | | 257 | 1.4 | |  | | 2 | 3.1 | | | 960 | 2.1 |  | |
| Grade 2 | 1390 | 52.7 | | 143337 | 48.3 | |  | | 162 | 43.0 | | 17244 | 39.9 | |  | | 4 | 13.8 | | 4166 | 22.5 | |  | | 10 | 15.4 | | | 7828 | 17.2 |  | |
| Grade 3 | 786 | 29.8 | | 55215 | 18.6 | |  | | 184 | 48.8 | | 20663 | 47.8 | |  | | 17 | 58.6 | | 12514 | 67.5 | |  | | 42 | 64.6 | | | 33999 | 74.9 |  | |
| Grade 4 | 4 | 0.2 | | 414 | 0.1 | |  | | 0 | 0.0 | | 158 | 0.4 | |  | | 0 | 0.0 | | 134 | 0.7 | |  | | 2 | 3.1 | | | 327 | 0.7 |  | |
| Unknown | 114 | 4.3 | | 12212 | 4.1 | |  | | 23 | 6.1 | | 2430 | 5.6 | |  | | 6 | 20.7 | | 1455 | 7.9 | |  | | 9 | 13.8 | | | 2286 | 5.0 |  | |
| T |  |  | |  |  | | <0.0001 | |  |  | |  |  | | <0.0001 | |  |  | |  |  | | <0.0001 | |  |  | | |  |  | <0.0001 | |
| T0/Tis | 27 | 1.0 | | 3510 | 1.2 | |  | | 4 | 1.1 | | 1028 | 2.4 | |  | | 2 | 6.9 | | 903 | 4.9 | |  | | 4 | 6.2 | | | 615 | 1.4 |  | |
| T1 | 1183 | 44.8 | | 183989 | 61.9 | |  | | 119 | 31.6 | | 19681.0 | 45.5 | |  | | 8 | 27.6 | | 6889 | 37.2 | |  | | 16 | 24.6 | | | 18807 | 41.4 |  | |
| T2 | 1059 | 40.1 | | 78644 | 26.5 | |  | | 180 | 47.7 | | 15265 | 35.3 | |  | | 8 | 27.6 | | 6412 | 34.6 | |  | | 33 | 50.8 | | | 17832 | 39.3 |  | |
| T3 | 82 | 3.1 | | 15465 | 5.2 | |  | | 17 | 4.5 | | 3257 | 7.5 | |  | | 2 | 6.9 | | 1741 | 9.4 | |  | | 1 | 1.5 | | | 3795 | 8.4 |  | |
| T4 | 221 | 8.4 | | 9029 | 3.0 | |  | | 46 | 12.2 | | 2723 | 6.3 | |  | | 2 | 6.9 | | 1921 | 10.4 | |  | | 4 | 6.2 | | | 3168 | 7.0 |  | |
| TX | 68 | 2.6 | | 6431 | 2.2 | |  | | 11 | 2.9 | | 1282 | 3.0 | |  | | 7 | 24.1 | | 660 | 3.6 | |  | | 7 | 10.8 | | | 1183 | 2.6 |  | |
| N |  |  | |  |  | | <0.0001 | |  |  | |  |  | | <0.0001 | |  |  | |  |  | | 0.186 | |  |  | | |  |  | 0.069 | |
| N0/N1mi | 1658 | 62.8 | | 221579 | 74.6 | |  | | 189 | 50.1 | | 27014 | 62.5 | |  | | 11 | 38.0 | | 10523 | 56.8 | |  | | 42 | 64.6 | | | 30214 | 66.6 |  | |
| N1 | 603 | 22.8 | | 50181 | 16.9 | |  | | 126 | 33.4 | | 10740 | 24.8 | |  | | 12 | 41.4 | | 5137 | 27.7 | |  | | 11 | 16.9 | | | 9580 | 21.1 |  | |
| N2 | 229 | 8.7 | | 13453 | 4.5 | |  | | 32 | 8.5 | | 2868 | 6.6 | |  | | 1 | 3.4 | | 1348 | 7.3 | |  | | 4 | 6.2 | | | 2683 | 5.9 |  | |
| N3 | 119 | 4.5 | | 7703 | 2.6 | |  | | 24 | 6.4 | | 1884 | 4.4 | |  | | 4 | 13.8 | | 1182 | 6.4 | |  | | 4 | 6.2 | | | 2200 | 4.8 |  | |
| NX | 31 | 1.2 | | 4152 | 1.4 | |  | | 6 | 1.6 | | 730 | 1.7 | |  | | 1 | 3.4 | | 336 | 1.8 | |  | | 4 | 6.2 | | | 723 | 1.6 |  | |
| M |  |  | |  |  | | <0.0001 | |  |  | |  |  | | 0.002 | |  |  | |  |  | | 0.011 | |  |  | | |  |  | <0.0001 | |
| M0 | 2467 | 93.4 | | 284329 | 95.7 | |  | | 331 | 87.8 | | 39821 | 92.1 | |  | | 22.0 | 75.9 | | 16680 | 90.0 | |  | | 43 | 66.2 | | | 42515 | 93.6 |  | |
| M1 | 173 | 6.6 | | 12739 | 4.3 | |  | | 46 | 12.2 | | 3415 | 7.9 | |  | | 7.0 | 24.1 | | 1846 | 10.0 | |  | | 22 | 33.8 | | | 2885 | 6.4 |  | |
| ER |  |  | |  |  | | <0.0001 | |  |  | |  |  | | 0.019 | |  |  | |  |  | |  | |  |  | | |  |  |  | |
| Positive | 2633 | 99.7 | | 294055 | 99.0 | |  | | 375 | 99.5 | | 41941 | 97.0 | |  | |  |  | |  |  | |  | |  |  | | |  |  |  | |
| Negative | 6 | 0.2 | | 2909 | 1.0 | |  | | 2 | 0.5 | | 1247 | 2.9 | |  | |  |  | |  |  | |  | |  |  | | |  |  |  | |
| Borderline/Unknown | 1 | <0.01 | | 104 | <0.01 | |  | | 0 | 0.0 | | 48 | 0.1 | |  | |  |  | |  |  | |  | |  |  | | |  |  |  | |
| PgR |  |  | |  |  | | <0.0001 | |  |  | |  |  | | <0.0001 | |  |  | |  |  | |  | |  |  | | |  |  |  | |
| Positive | 2464 | 93.3 | | 260078 | 87.5 | |  | | 323 | 85.7 | | 31591 | 73.1 | |  | |  |  | |  |  | |  | |  |  | | |  |  |  | |
| Negative | 172 | 6.5 | | 36641 | 12.3 | |  | | 54 | 14.3 | | 11555 | 26.7 | |  | |  |  | |  |  | |  | |  |  | | |  |  |  | |
| Borderline/Unknown | 4 | 0.2 | | 349 | 0.1 | |  | | 0 | 0.0 | | 90 | 0.2 | |  | |  |  | |  |  | |  | |  |  | | |  |  |  | |
| HER2 |  |  | |  |  | |  | |  |  | |  |  | |  | |  |  | |  |  | |  | |  |  | | |  |  |  | |
| Positive |  |  | |  |  | |  | |  |  | |  |  | |  | |  |  | |  |  | |  | |  |  | | |  |  |  | |
| Negative |  |  | |  |  | |  | |  |  | |  |  | |  | |  |  | |  |  | |  | |  |  | | |  |  |  | |
| Surgery |  |  | |  |  | | 0.026 | |  |  | |  |  | | 0.186 | |  |  | |  |  | | <0.0001 | |  |  | | |  |  | <0.0001 | |
| Yes | 2401 | 90.9 | | 273656 | 92.1 | |  | | 323 | 85.7 | | 38008 | 87.9 | |  | | 18 | 62.1 | | 15840 | 85.5 | |  | | 42 | 64.6 | | | 40628 | 89.5 |  | |
| No/Unknown | 239 | 9.1 | | 23412 | 7.9 | |  | | 54 | 14.3 | | 5228.0 | 12.1 | |  | | 11 | 37.9 | | 2686 | 14.5 | |  | | 23 | 35.4 | | | 4772 | 10.5 |  | |
| Radiotherapy |  |  | |  |  | | <0.0001 | |  |  | |  |  | | <0.0001 | |  |  | |  |  | | 0.572 | |  |  | | |  |  | 0.695 | |
| Yes | 732 | 27.7 | | 149598 | 50.4 | |  | | 103 | 27.3 | | 19273 | 44.6 | |  | | 10 | 34.5 | | 10850 | 58.6 | |  | | 28 | 43.1 | | | 24743 | 54.5 |  | |
| No/Unknown | 1908 | 72.3 | | 147470 | 49.6 | |  | | 274 | 72.7 | | 23963 | 55.4 | |  | | 19 | 65.5 | | 7676 | 41.4 | |  | | 37 | 56.9 | | | 20657 | 45.5 |  | |
| Chemotherapy |  |  | |  |  | | <0.0001 | |  |  | |  |  | | 0.283 | |  |  | |  |  | | 0.604 | |  |  | | |  |  | 0.058 | |
| Yes | 847 | 32.1 | | 84234 | 28.4 | |  | | 257 | 68.2 | | 30568 | 70.7 | |  | | 23 | 79.3 | | 13921 | 75.1 | |  | | 40 | 61.5 | | | 32734 | 72.1 |  | |
| No/Unknown | 1793 | 67.9 | | 212834 | 71.6 | |  | | 120 | 31.8 | | 12668 | 29.3 | |  | | 6 | 20.7 | | 4605 | 24.9 | |  | | 25 | 38.5 | | | 12666 | 27.9 |  | |

MBC=male breast cancer; FBC=female breast cancer; DC=ductal carcinoma; LC=lobular carcinoma; ER=estrogen receptor; PgR=progesterone receptor; HER2=human epidermal growth factor receptor 2

**Supplementary Table2. Baseline characteristics of early disease from MBC and FBC.**

| **Characteristics** | **Male (N=2863)** | | **Female (N=383345)** | | ***P* value** |
| --- | --- | --- | --- | --- | --- |
|  | N | Percent (%) | N | Percent (%) |  |
| Age at diagnosis, median, y | 67.37 |  | 61.71 |  | <0.0001 |
| Age group at diagnosis, y |  |  |  |  | <0.0001 |
| <40 | 45 | 1.6 | 17728 | 4.6 |  |
| 40-49 | 184 | 6.4 | 58492 | 15.3 |  |
| 50-59 | 500 | 17.5 | 90916 | 23.7 |  |
| 60-69 | 875 | 30.6 | 105231 | 27.5 |  |
| 70-79 | 757 | 26.4 | 71363 | 18.6 |  |
| ≥80 | 502 | 17.5 | 39615 | 10.3 |  |
| Race |  |  |  |  | <0.0001 |
| White | 2288 | 79.9 | 303853 | 79.3 |  |
| Black | 412 | 14.4 | 41446 | 10.8 |  |
| Other | 147 | 5.1 | 35349 | 9.2 |  |
| Unknown | 16 | 0.6 | 2697 | 0.7 |  |
| Primary Site |  |  |  |  | <0.0001 |
| Upper-outer | 336 | 11.7 | 130813 | 34.1 |  |
| Lower-outer | 107 | 3.7 | 29139 | 7.6 |  |
| Upper-inner | 125 | 4.4 | 48242 | 12.6 |  |
| Lower-inner | 45 | 1.6 | 21757 | 5.7 |  |
| Central portion | 1224 | 42.8 | 17846 | 4.7 |  |
| Nipple | 147 | 5.1 | 1423 | 0.4 |  |
| Axillary tail | 3 | 0.1 | 2011 | 0.5 |  |
| Overlapping | 446 | 15.6 | 88044 | 23.0 |  |
| Unknown | 430 | 15.0 | 44070 | 11.5 |  |
| Laterality |  |  |  |  | 0.028 |
| Right | 1343 | 47.0 | 189066 | 49.4 |  |
| Left | 1517 | 53.0 | 194083 | 50.6 |  |
| Bilateral | 0 | 0.0 | 28 | <0.01 |  |
| Unknown | 3 | 0.1 | 168 | <0.01 |  |
| Histologic type |  |  |  |  | <0.0001 |
| DC | 2587 | 90.4 | 301742 | 78.7 |  |
| LC | 87 | 3.0 | 60351 | 15.7 |  |
| Others | 189 | 6.6 | 21252 | 5.5 |  |
| Grade |  |  |  |  | <0.0001 |
| Grade 1 | 348 | 12.2 | 88403 | 23.1 |  |
| Grade 2 | 1471 | 51.4 | 165378 | 43.1 |  |
| Grade 3 | 931 | 32.5 | 114175 | 29.8 |  |
| Grade 4 | 4 | 0.1 | 912 | 0.2 |  |
| Unknown | 109 | 3.8 | 14477 | 3.8 |  |
| T |  |  |  |  | <0.0001 |
| T0/Tis | 28 | 1.0 | 5678 | 1.5 |  |
| T1 | 1302 | 45.5 | 226842 | 59.2 |  |
| T2 | 1197 | 41.8 | 112277 | 29.3 |  |
| T3 | 76 | 2.7 | 21194 | 5.5 |  |
| T4 | 196 | 6.8 | 10479 | 2.7 |  |
| TX | 64 | 2.2 | 6875 | 1.8 |  |
| N |  |  |  |  |  |
| N0/N1mi | 1839 | 64.2 | 284155 | 74.1 | <0.0001 |
| N1 | 644 | 22.5 | 67027 | 17.5 |  |
| N2 | 231 | 8.1 | 18089 | 4.7 |  |
| N3 | 120 | 4.2 | 9958 | 2.6 |  |
| NX | 29 | 1.0 | 4116 | 1.1 |  |
| Subtype |  |  |  |  | <0.0001 |
| HR+/HER2- | 2467 | 86.2 | 284329 | 74.2 |  |
| HR+/HER2+ | 331 | 11.6 | 39821 | 10.4 |  |
| HR-/HER2+ | 22 | 0.8 | 16680 | 4.4 |  |
| HR-/HER2- | 43 | 1.5 | 42515 | 11.1 |  |
| ER |  |  |  |  | <0.0001 |
| Positive | 2793 | 97.6 | 320180 | 83.5 |  |
| Negative | 70 | 2.4 | 63022 | 16.4 |  |
| Borderline/Unknown | 0 | 0.0 | 143 | <0.01 |  |
| PgR |  |  |  |  | <0.0001 |
| Positive | 2594 | 90.6 | 279044 | 72.8 |  |
| Negative | 265 | 9.3 | 103903 | 27.1 |  |
| Borderline/Unknown | 4 | 0.1 | 398 | 0.1 |  |
| HER2 |  |  |  |  | <0.0001 |
| Positive | 353 | 12.3 | 56501 | 14.7 |  |
| Negative | 2510 | 87.7 | 326844 | 85.3 |  |
| Surgery |  |  |  |  | 0.554 |
| Yes | 2695 | 94.1 | 361830 | 94.4 |  |
| No/Unknown | 168 | 5.9 | 21515 | 5.6 |  |
| Radiotherapy |  |  |  |  | <0.0001 |
| Yes | 789 | 27.6 | 190627 | 49.7 |  |
| No/Unknown | 2074 | 72.4 | 192718 | 50.3 |  |
| Chemotherapy |  |  |  |  | 0.002 |
| Yes | 1037 | 36.2 | 149785 | 39.1 |  |
| No/Unknown | 1826 | 63.8 | 233560 | 60.9 |  |

MBC=male breast cancer; FBC=female breast cancer; DC=ductal carcinoma; LC=lobular carcinoma; ER=estrogen receptor; PgR=progesterone receptor; HER2=human epidermal growth factor receptor 2

**Supplementary Table3. Baseline characteristics of metastatic disease from MBC and FBC.**

| **Characteristics** | **Male (N=248)** | | **Female (N=20885)** | | ***P* value** |
| --- | --- | --- | --- | --- | --- |
|  | N | Percent (%) | N | Percent (%) |  |
| Age at diagnosis, median, y | 64.18 |  | 61.53 |  | 0.003 |
| Age group at diagnosis, y |  |  |  |  | 0.002 |
| <40 | 9 | 3.6 | 1394 | 6.7 |  |
| 40-49 | 26 | 10.5 | 2748 | 13.2 |  |
| 50-59 | 45 | 18.1 | 5218 | 25.0 |  |
| 60-69 | 77 | 31.0 | 5500 | 26.3 |  |
| 70-79 | 66 | 26.6 | 3537 | 16.9 |  |
| ≥80 | 25 | 10.1 | 2488 | 11.9 |  |
| Race |  |  |  |  | 0.290 |
| White | 15691 | 75.1 | 181 | 73.0 |  |
| Black | 51 | 20.6 | 3444 | 16.5 |  |
| Other | 15 | 6.0 | 1675 | 8.0 |  |
| Unknown | 1 | 0.4 | 75 | 0.4 |  |
| Primary Site |  |  |  |  | <0.0001 |
| Upper-outer | 248 | 9.7 | 4974 | 23.8 |  |
| Lower-outer | 2 | 0.8 | 1120 | 5.4 |  |
| Upper-inner | 3 | 1.2 | 1313 | 6.3 |  |
| Lower-inner | 6 | 2.4 | 731 | 3.5 |  |
| Central portion | 82 | 33.1 | 1270 | 6.1 |  |
| Nipple | 11 | 4.4 | 72 | 0.3 |  |
| Axillary tail | 2 | 0.8 | 183 | 0.9 |  |
| Overlapping | 36 | 14.5 | 4135 | 19.8 |  |
| Unknown | 82 | 33.1 | 7087 | 33.9 |  |
| Laterality |  |  |  |  | 0.170 |
| Right | 109 | 44.0 | 9866 | 47.2 |  |
| Left | 134 | 54.0 | 10277 | 49.2 |  |
| Bilateral | 0 | 0.0 | 97 | 0.5 |  |
| Unknown | 5 | 2.0 | 645 | 3.1 |  |
| Histologic type |  |  |  |  | <0.0001 |
| DC | 208 | 83.9 | 14749 | 70.6 |  |
| LC | 8 | 3.2 | 3332 | 16.0 |  |
| Others | 32 | 12.9 | 2804 | 13.4 |  |
| Grade |  |  |  |  | <0.0001 |
| Grade 1 | 10 | 4.0 | 1445 | 6.9 |  |
| Grade 2 | 95 | 38.3 | 7197 | 34.5 |  |
| Grade 3 | 98 | 39.5 | 8216 | 39.3 |  |
| Grade 4 | 2 | 0.8 | 121 | 0.6 |  |
| Unknown | 3906 | 18.7 | 43 | 17.3 |  |
| T |  |  |  |  | 0.077 |
| T0 | 9 | 3.6 | 376 | 1.8 |  |
| Tis | 0 | 0.0 | 2 | <0.01 |  |
| T1 | 24 | 9.7 | 2524 | 12.1 |  |
| T2 | 83 | 33.5 | 5876 | 28.1 |  |
| T3 | 26 | 10.5 | 3064 | 14.7 |  |
| T4 | 77 | 31.0 | 6362 | 30.5 |  |
| TX | 29 | 11.7 | 2681 | 12.8 |  |
| N |  |  |  |  | 0.219 |
| N0 | 58 | 23.4 | 4979 | 23.8 |  |
| N1mi | 3 | 1.2 | 196 | 0.9 |  |
| N1 | 108 | 43.5 | 8611 | 41.2 |  |
| N2 | 35 | 14.1 | 2263 | 10.8 |  |
| N3 | 31 | 12.5 | 3011 | 14.4 |  |
| NX | 13 | 5.2 | 1825 | 8.7 |  |
| Subtype |  |  |  |  | <0.0001 |
| HR+/HER2- | 173 | 69.8 | 12739 | 61.0 |  |
| HR+/HER2+ | 46 | 18.5 | 3415 | 16.4 |  |
| HR-/HER2+ | 7 | 2.8 | 1846 | 8.8 |  |
| HR-/HER2- | 22 | 8.9 | 2885 | 13.8 |  |
| ER |  |  |  |  | <0.0001 |
| Positive | 215 | 86.7 | 15816 | 75.7 |  |
| Negative | 32 | 12.9 | 5055 | 24.2 |  |
| Borderline/Unknown | 1 | 0.4 | 14 | 0.1 |  |
| PgR |  |  |  |  | <0.0001 |
| Positive | 193 | 77.8 | 12625 | 60.5 |  |
| Negative | 55 | 22.2 | 8119 | 38.9 |  |
| Borderline/Unknown | 0 | 0.0 | 141 | 0.7 |  |
| HER2 |  |  |  |  | 0.187 |
| Positive | 53 | 21.4 | 5261 | 25.2 |  |
| Negative | 195 | 78.6 | 15624 | 74.8 |  |
| Surgery |  |  |  |  | 0.059 |
| Yes | 89 | 35.9 | 6302 | 30.2 |  |
| No/Unknown | 159 | 64.1 | 14583 | 69.8 |  |
| Radiotherapy |  |  |  |  | 0.451 |
| Yes | 84 | 33.9 | 6577 | 31.5 |  |
| No/Unknown | 164 | 66.1 | 14308 | 68.5 |  |
| Chemotherapy |  |  |  |  | 0.275 |
| Yes | 130 | 52.4 | 11672 | 55.9 |  |
| No/Unknown | 118 | 47.6 | 9213 | 44.1 |  |
| Bone |  |  |  |  | 0.052 |
| Yes | 178 | 71.8 | 13515 | 64.7 |  |
| No | 65 | 26.2 | 6971 | 33.4 |  |
| Unknown | 5 | 2.0 | 399 | 1.9 |  |
| Liver |  |  |  |  | <0.0001 |
| Yes | 26 | 10.5 | 5126 | 24.5 |  |
| No | 215 | 86.7 | 15171 | 72.6 |  |
| Unknown | 7 | 2.8 | 588 | 2.8 |  |
| Lung |  |  |  |  | 0.033 |
| Yes | 93 | 37.5 | 6226 | 29.8 |  |
| No | 146 | 58.9 | 13926 | 66.7 |  |
| Unknown | 9 | 3.6 | 733 | 3.5 |  |
| Brain |  |  |  |  | 0.871 |
| Yes | 19 | 7.7 | 1441 | 6.9 |  |
| No | 221 | 89.1 | 18704 | 89.6 |  |
| Unknown | 8 | 3.2 | 740 | 3.5 |  |
| Visceral metastasis |  |  |  |  | 0.454 |
| Yes | 110 | 44.4 | 10042 | 48.1 |  |
| No | 127 | 51.2 | 10090 | 48.3 |  |
| Unknown | 11 | 4.4 | 753 | 3.6 |  |

MBC=male breast cancer; FBC=female breast cancer; DC=ductal carcinoma; LC=lobular carcinoma; ER=estrogen receptor; PgR=progesterone receptor; HER2=human epidermal growth factor receptor 2

**Supplementary Table4. Metastatic patterns in MBC and FBC.**

| **Features** | **Male (n=248)** | | **Female (N=20885)** | | ***P* value** |
| --- | --- | --- | --- | --- | --- |
|  | N | Percent (%) | N | Percent (%) |  |
| One site |  |  |  |  |  |
| Bone | 95 | 38.3 | 7473 | 35.8 | 0.682 |
| Liver | 3 | 1.2 | 1358 | 6.5 | <0.0001 |
| Lung | 25 | 10.1 | 1993 | 9.5 | 0.960 |
| Brain | 2 | 0.8 | 243 | 1.2 | 0.858 |
| Two sites |  |  |  |  |  |
| Bone and liver | 7 | 2.8 | 1595 | 7.6 | 0.005 |
| Bone and lung | 43 | 17.3 | 1895 | 9.1 | <0.0001 |
| Bone and brain | 4 | 1.6 | 309 | 1.5 | 0.986 |
| Liver and lung | 2 | 0.8 | 460 | 2.2 | 0.230 |
| Liver and brain | 0 | 0.0 | 49 | 0.2 | 0.560 |
| Lung and brain | 0 | 0.0 | 134 | 0.6 | 0.204 |
| Three sites |  |  |  |  |  |
| Bone, liver, and lung | 9 | 3.6 | 958 | 4.6 | 0.756 |
| Bone, liver, and brain | 1 | 0.4 | 125 | 0.6 | 0.915 |
| Bone, lung, and brain | 9 | 3.6 | 208 | 1.0 | 0.006 |
| Liver, lung, and brain | 0 | 0.0 | 56 | 0.3 | 0.516 |
| Four sites |  |  |  |  |  |
| Bone, liver, lung, and brain | 3 | 1.2 | 235 | 1.1 | 0.992 |

MBC=male breast cancer; FBC=female breast cancer;

**Supplementary Table5. Comparative OS and BCSS of MBC and FBC.**

| Characteristics | OS | | | | | BCSS | | | | |
| --- | --- | --- | --- | --- | --- | --- | --- | --- | --- | --- |
|  | MBC | | FBC | | *P* value | MBC | | FBC | | *P* value |
|  | Median (95%CI) | Mean (95%CI) | Median (95%CI) | Mean (95%CI) |  | Median (95%CI) | Mean (95%CI) | Median (95%CI) | Mean (95%CI) |  |
| Overall | NA | 65.5 (64.4-66.7) | NA | 72.7 (72.7-72.8) | <0.0001 | NA | 75.4 (74.4-76.3) | NA | 77.8 (77.7-77.8) | <0.0001 |
| Age |  |  |  |  |  |  |  |  |  |  |
| Young | NA | 73.1 (66.1-81.1) | NA | 74.4 (74.1-74.8) | 0.781 | NA | 74.7 (68.2-81.2) | NA | 75.7 (75.3-76.0) | 0.873 |
| Elderly | 78.0 (NA) | 60.0 (58.2-61.9) | NA | 65.3 (65.1-65.5) | <0.0001 | NA | 76.1 (74.6-77.7) | NA | 75.9 (75.8-76.1) | 0.591 |
| Subtype |  |  |  |  |  |  |  |  |  |  |
| HR+/HER2- | NA | 66.6 (65.4-67.9) | NA | 74.1 (74.0-74.2) | <0.0001 | NA | 76.7 (75.8-77.7) | NA | 79.1 (79.0-79.2) | <0.0001 |
| HR+/HER2+ | NA | 62.2 (58.6-65.9) | NA | 73.09 (72.8-73.4) | <0.0001 | NA | 70.3 (66.9-73.6) | NA | 77.4 (77.2-77.6) | <0.0001 |
| HR-/HER2+ | 78.0 (NA) | 59.3 (46.5-72.1) | NA | 69.4 (68.9-69.9) | 0.027 | 78.0 (NA) | 68.9 (57.9-79.9) | NA | 73.9 (73.6-74.3) | 0.253 |
| HR-/HER2- | 18 (0.9-36.0) | 32.1 (24.7-39.5) | NA | 65.1 (64.8-65.4) | <0.0001 | NA | 36.6 (28.7-44.6) | NA | 70.6 (70.4-70.9) | <0.0001 |

OS=overall survival; BCSS=breast cancer-specific survival; MBC=male breast cancer; FBC=female breast cancer;

**Supplementary Table6. Comparative OS and BCSS of MBC and FBC.**

| Characteristics | OS | | | | | BCSS | | | | |
| --- | --- | --- | --- | --- | --- | --- | --- | --- | --- | --- |
|  | MBC | | FBC | | *P* value | MBC | | FBC | | *P* value |
|  | Median (95%CI) | Mean (95%CI) | Median (95%CI) | Mean (95%CI) |  | Median (95%CI) | Mean (95%CI) | Median (95%CI) | Mean (95%CI) |  |
| Overall | NA | 65.6 (64.5-66.8) | NA | 68.3 (67.5-69.1) | <0.0001 | NA | 75.4 (74.5-76.4) | NA | 76.2 (75.5-76.8) | 0.256 |
| Age |  |  |  |  |  |  |  |  |  |  |
| Young | NA | 73.1 (66.1-80.1) | NA | 69.9 (65.2-74.8) | 0.740 | NA | 74.7 (68.2-81.2) | NA | 71.7 (67.1-76.3) | 0.673 |
| Elderly | NA | 59.6 (57.7-61.6) | NA | 60.9 (59.6-62.3) | 0.291 | NA | 76.2 (74.7-77.7) | NA | 74.5 (73.4-75.6) | 0.055 |
| Subtype |  |  |  |  |  |  |  |  |  |  |
| HR+/HER2- | NA | 66.7 (65.5-67.9) | NA | 68.5 (67.6-69.3) | 0.026 | NA | 76.8 (75.9-77.7) | NA | 76.5 (75.8-77.2) | 0.460 |
| HR+/HER2+ | NA | 62.2 (58.6-65.9) | NA | 67.5 (65.2-69.9) | 0.024 | NA | 70.3 (66.9-73.6) | NA | 74.7 (72.7-76.7) | 0.057 |
| HR-/HER2+ | 78.0 (NA) | 59.3 (46.5-72.1) | NA | 71.6 (65.1-78.1) | 0.068 | 78.0 (NA) | 68.9 (57.9-79.9) | NA | 75.7 (70.1-81.2) | 0.267 |
| HR-/HER2- | 18.0 (0.2-36.0) | 32.1 (24.7-39.5) | NA | 63.3 (58.0-68.7) | <0.0001 | NA | 36.6 (28.7-44.6) | NA | 71.2 (66.5-75.9) | <0.0001 |

OS=overall survival; BCSS=breast cancer-specific survival; MBC=male breast cancer; FBC=female breast cancer;

**Supplementary Table7. The OS and BCSS of MBC stratified by clinicopathological characteristics.**

| Characteristics | OS | | | BCSS | | |
| --- | --- | --- | --- | --- | --- | --- |
|  | Median (95%CI) | Mean (95%CI) | *P* value | Median (95%CI) | Mean (95%CI) | *P* value |
| Age group at diagnosis, y |  |  | <0.0001 |  |  | 0.164 |
| <40 | NA | 73.1 (66.1-80.1) |  | NA | 74.7 (68.2-81.2) |  |
| 40-49 | NA | 70.6 (66.7-74.6) |  | NA | 72.3 (68.6-76.1) |  |
| 50-59 | NA | 70.4 (67.9-72.9) |  | NA | 75.4 (73.2-77.6) |  |
| 60-69 | NA | 69.3 (67.4-71.2) |  | NA | 75.2 (73.5-76.8) |  |
| 70-79 | NA | 65.7 (63.5-67.9) |  | NA | 77.2 (75.6-78.9) |  |
| ≥80 | 53.0 (46.8-59.2) | 51.3 (48.3-54.3) |  | NA | 74.4 (71.5-77.3) |  |
| Race |  |  | 0.002 |  |  | <0.0001 |
| White | NA | 66.2 (64.9-67.5) |  | NA | 76.3 (75.3-77.3) |  |
| Black | 78.0 (NA) | 61.1 (57.9-64.2) |  | NA | 69.6 (66.5-72.6) |  |
| Others | NA | 68.5 (63.5-73.5) |  | NA | 77.3 (73.8-80.8) |  |
| Primary Site |  |  | <0.0001 |  |  | <0.0001 |
| Upper-outer | NA | 69.0 (65.7-73.3) |  | NA | 77.4 (74.8-79.9) |  |
| Lower-outer | NA | 71.8 (67.2-76.5) |  | NA | 78.3 (75.0-81.6) |  |
| Upper-inner | NA | 68.3 (62.5-74.1) |  | NA | 76.7 (72.2-81.1) |  |
| Lower-inner | NA | 65.9 (57.1-74.8) |  | NA | 77.3 (71.1-83.5) |  |
| Central | NA | 66.2 (64.5-67.9) |  | NA | 76.7 (75.3-78.0) |  |
| Overlapping | NA | 68.2 (65.3-71.1) |  | NA | 75.6 (73.3-78.0) |  |
| Others | NA | 64.9 (60.0-69.9) |  | NA | 77.8 (74.4-81.2) |  |
| Unknown | 70.0 (NA) | 57.9 (54.8-60.9) |  | NA | 68.3 (65.3-71.3) |  |
| Laterality |  |  | 0.046 |  |  | 0.022 |
| Right | NA | 65.8 (64.1-67.5) |  | NA | 76.1 (74.8-77.4) |  |
| Left | NA | 65.5 (63.9-67.1) |  | NA | 74.9 (73.5-76.2) |  |
| Others | 35.0 (8.1-61.9) | 38.1 (15.4-60.8) |  | NA | 46.3 (17.6-74.9) |  |
| Histologic type |  |  | 0.006 |  |  | 0.001 |
| DC | NA | 65.9 (64.7-67.2) |  | NA | 75.7 (74.7-76.7) |  |
| LC | NA | 69.0 (62.2-75.9) |  | NA | 78.2 (72.7-83.7) |  |
| Others | 78.0 (NA) | 58.9 (54.3-63.5) |  | NA | 69.3 (64.9-73.6) |  |
| Grade |  |  | <0.0001 |  |  | <0.0001 |
| Grade 1 | NA | 71.4 (68.4-74.4) |  | NA | 79.7 (77.8-81.6) |  |
| Grade 2 | NA | 68.6 (66.9-70.1) |  | NA | 77.9 (76.9-79.1) |  |
| Grade 3 | 78.0 (NA) | 61.7 (59.6-63.7) |  | NA | 72.3 (70.4-74.2) |  |
| Grade 4 | 28.0 (NA) | 28.3 (17.9-38.7) |  | 32.0 (23.4-40.6) | 32.4 (23.5-41.3) |  |
| Unknown | 54.0 (34.6-73.5) | 50.5 (44.4-56.2) |  | NA | 59.4 (52.9-65.9) |  |
| T |  |  | <0.0001 |  |  | <0.0001 |
| T0/Tis/T1 | NA | 72.1 (70.6-73.6) |  | NA | 80.3 (79.4-81.1) |  |
| T2 | NA | 63.6 (61.7-65.5) |  | NA | 73.9 (72.4-75.4) |  |
| T3 | 59.0 (49.4-68.6) | 50.9 (44.4-57.6) |  | 62.0 (NA) | 73.9 (72.4-75.5) |  |
| T4 | 49.0 (38.5-59.5) | 48.4 (44.4-52.5) |  | 68.0 (60.7-75.3) | 73.9 (72.4-75.6) |  |
| TX | 66.0 (36.3-95.7) | 51.9 (44.8-58.9) |  | NA | 73.9 (72.4-75.7) |  |
| N |  |  | <0.0001 |  |  | <0.0001 |
| N0/N1mi | NA | 68.8 (67.5-70.2) |  | NA | 78.8 (77.9-79.8) |  |
| N1 | NA | 61.7 (59.2-64.3) |  | NA | 71.1 (68.8-73.4) |  |
| N2 | 76.0 (65.9-86.1) | 60.2 (56.1-64.3) |  | NA | 70.9 (66.9-74.8) |  |
| N3 | 67.0 (52.9-81.0) | 60.1 (54.9-65.3) |  | 39.0 (17.0-60.9) | 64.9 (59.5-70.2) |  |
| NX | 27.0 (9.4-44.6) | 31.8 (22.5-41.2) |  | NA | 40.4 (25.9-54.9) |  |
| M |  |  | <0.0001 |  |  | <0.0001 |
| M0 | NA | 68.2 (67.1-69.3) |  | NA | 78.3 (77.4-79.1) |  |
| M1 | 31.0 (23.8-38.2) | 34.7 (30.7-38.7) |  | 36.0 (23.7-48.3) | 39.3 (34.5-44.0) |  |
| Subtype |  |  | <0.0001 |  |  | <0.0001 |
| HR+/HER2- | NA | 66.6 (65.4-67.9) |  | NA | 76.7 (75.8-77.7) |  |
| HR+/HER2+ | NA | 62.2 (58.6-65.9) |  | NA | 70.3 (66.9-73.6) |  |
| HR-/HER2+ | 78.0 (NA) | 59.3 (46.5-72.1) |  | 78.0 (NA) | 68.9 (57.9-79.9) |  |
| HR-/HER2- | 18.0 (0.2-36.0) | 32.1 (24.7-39.5) |  | NA | 36.6 (28.7-44.6) |  |
| Surgery |  |  | <0.0001 |  |  | <0.0001 |
| Yes | NA | 68.6 (67.5-69.8) |  | NA | 77.6 (76.8-78.5) |  |
| No/Unknown | 31.0 (26.9-35.1) | 35.4 (31.3-39.4) |  | 49.0 (30.6-67.4) | 46.7 (41.3-51.9) |  |
| Radiotherapy |  |  | 0.471 |  |  | 0.120 |
| Yes | NA | 65.9 (63.7-68.2) |  | NA | 73.9 (72.0-75.9) |  |
| No/Unknown | NA | 65.4 (64.0-66.7) |  | NA | 75.9 (74.8-76.9) |  |
| Chemotherapy |  |  | 0.004 |  |  | 0.007 |
| Yes | NA | 67.7 (65.9-69.5) |  | NA | 76.5 (75.3-77.6) |  |
| No/Unknown | NA | 64.3 (62.8-65.8) |  | NA | 73.6 (72.0-75.3) |  |
| Visceral metastasis |  |  |  |  |  | <0.0001 |
| No |  | 67.1 (65.9-68.2) |  | NA | 76.8 (75.9-77.8) |  |
| Yes | 20.0 (11.3-28.7) | 31.0 (25.1-36.9) |  | 29.0 (17.9-40.0) | 36.3 (29.1-43.4) |  |
| Unknown | 37.0 (0.1-78.8) | 48.5 (37.9-58.9) |  | NA | 65.8 (54.4-77.2) |  |
| Bone |  |  | <0.0001 |  |  | <0.0001 |
| No | NA | 67.7 (66.5-68.8) |  | NA | 77.5 (76.7-78.4) |  |
| Yes | 29.0 (21.6-36.4) | 32.6 (28.0-37.2) |  | 31.0 (19.4-42.6) | 36.1 (30.6-41.6) |  |
| Unknown | 37.0 (0.7-77.6) | 49.9 (39.3-60.6) |  | NA | 69.0 (58.7-79.3) |  |
| Liver |  |  | <0.0001 |  |  | <0.0001 |
| No | NA | 66.1 (64.9-67.3) |  | NA | 75.8 (74.9-76.8) |  |
| Yes | 14.0 (7.9-20.1) | 22.3 (13.8-30.7) |  | 14.0 (0.3-35.2) | 24.8 (15.1-36.5) |  |
| Unknown | 37.0 (29.7-44.3) | 46.7 (36.4-56.9) |  | NA | 63.3 (51.5-76.1) |  |
| Lung |  |  | <0.0001 |  |  | <0.0001 |
| No | NA | 66.9 (65.8-68.1) |  | NA | 76.7 (75.7-77.6) |  |
| Yes | 20.0 (10.9-29.1) | 32.6 (26.0-39.2) |  | 29.0 (16.8-41.2) | 38.4 (30.6-46.2) |  |
| Unknown | 37.0 (26.2-47.8) | 46.2 (36.5-55.9) |  | NA | 61.1 (50.2-72.1) |  |
| Brain |  |  | <0.0001 |  |  | <0.0001 |
| No | NA | 66.1 (64.9-67.2) |  | NA | 75.7 (74.8-76.6) |  |
| Yes | 8.0 (2.3-13.7) | 13.7 (6.8-20.6) |  | 10.0 (1.2-18.8) | 16.3 (8.2-24.4) |  |
| Unknown | 37.0 (31.0-42.9) | 47.1 (37.1-57.1) |  | NA | 63.3 (51.5-75.1) |  |

OS=overall survival; BCSS=breast cancer-specific survival; MBC=male breast cancer; FBC=female breast cancer; DC=ductal carcinoma; LC=lobular carcinoma; ER=estrogen receptor; PgR=progesterone receptor; HER2=human epidermal growth factor receptor 2

**Supplementary Table8. Risk factors for BCSS of MBC.**

| Characteristics | Univariate | | | Multivariate | | |
| --- | --- | --- | --- | --- | --- | --- |
|  | Hazard ratio | 95%CI | *P* value | Hazard ratio | 95%CI | *P* value |
| Age group at diagnosis, y |  |  | 0.173 |  |  |  |
| <40 | Reference |  |  |  |  |  |
| 40-49 | 1.40 | 0.49-4.00 | 0.535 |  |  |  |
| 50-59 | 0.95 | 0.34-2.67 | 0.929 |  |  |  |
| 60-69 | 1.01 | 0.37-2.75 | 0.993 |  |  |  |
| 70-79 | 0.73 | 0.26-2.04 | 0.549 |  |  |  |
| ≥80 | 1.13 | 0.39-3.21 | 0.818 |  |  |  |
| Race |  |  | <0.0001 |  |  | 0.001 |
| White | Reference |  |  | Reference |  |  |
| Black | 2.14 | 1.58-2.89 | <0.0001 | 1.76 | 1.27-2.44 | 0.001 |
| Others | 0.75 | 0.35-1.59 | 0.447 | 0.72 | 0.33-1.57 | 0.413 |
| Primary Site |  |  | <0.0001 |  |  |  |
| Upper-outer | Reference |  |  | Reference |  | 0.250 |
| Lower-outer | 0.78 | 0.29-2.11 | 0.626 | 0.75 | 0.27-2.07 | 0.572 |
| Upper-inner | 1.15 | 0.48-2.77 | 0.747 | 1.50 | 0.61-3.70 | 0.375 |
| Lower-inner | 1.24 | 0.36-4.20 | 0.733 | 1.32 | 0.37-4.64 | 0.668 |
| Central | 1.14 | 0.68-1.91 | 0.616 | 1.05 | 0.61-1.80 | 0.866 |
| Overlapping | 1.34 | 0.75-2.38 | 0.321 | 0.98 | 0.54-1.79 | 0.951 |
| Others | 0.94 | 0.41-2.16 | 0.878 | 0.87 | 0.37-2.04 | 0.746 |
| Unknown | 2.96 | 1.77-4.97 | <0.0001 | 1.58 | 0.91-2.76 | 0.108 |
| Laterality |  |  | 0.040 |  |  | 0.421 |
| Right | Reference |  |  | Reference |  |  |
| Left | 1.18 | 0.91-1.55 | 0.218 | 1.21 | 0.91-1.61 | 0.191 |
| Others | 5.35 | 1.32-21.71 | 0.019 | 0.98 | 0.21-4.68 | 0.983 |
| Histologic type |  |  | 0.002 |  |  | 0.473 |
| DC | Reference |  |  | Reference |  |  |
| LC | 0.51 | 4.45-16.68 | 0.250 | 0.48 | 0.15-1.57 | 0.226 |
| Others | 1.98 | 1.32-2.97 | 0.001 | 0.94 | 0.52-1.69 | 0.838 |
| Grade |  |  | <0.0001 |  |  | 0.001 |
| Grade 1 | Reference |  |  | Reference |  |  |
| Grade 2 | 1.46 | 0.79-2.69 | <0.0001 | 1.07 | 0.57-2.02 | 0.827 |
| Grade 3 | 3.32 | 1.82-6.04 | <0.0001 | 1.89 | 1.01-3.54 | 0.047 |
| Grade 4 | 18.93 | 5.33-67.26 | <0.0001 | 2.20 | 0.49-9.91 | 0.303 |
| Unknown | 8.61 | 4.45-16.68 | 0.192 | 2.57 | 1.23-5.39 | 0.012 |
| T |  |  | <0.0001 |  |  | <0.0001 |
| T0/Tis/T1 | Reference |  |  | Reference |  |  |
| T2 | 3.25 | 2.22-4.75 | <0.0001 | 2.58 | 1.73-3.84 | <0.0001 |
| T3 | 9.47 | 5.39-16.66 | <0.0001 | 5.11 | 2.79-9.38 | <0.0001 |
| T4 | 10.57 | 6.95-16.06 | <0.0001 | 2.87 | 1.71-4.81 | <0.0001 |
| TX | 8.93 | 5.03-15.86 | <0.0001 | 0.97 | 0.44-2.14 | 0.934 |
| N |  |  | <0.0001 |  |  | 0.006 |
| N0/N1mi | Reference |  |  | Reference |  |  |
| N1 | 3.03 | 2.22-4.15 | <0.0001 | 1.67 | 1.17-2.39 | 0.005 |
| N2 | 2.93 | 1.89-4.52 | <0.0001 | 1.85 | 1.13-3.02 | 0.014 |
| N3 | 4.51 | 2.89-7.04 | <0.0001 | 1.60 | 0.95-2.69 | 0.078 |
| NX | 14.68 | 7.32-29.42 | <0.0001 | 3.87 | 1.53-9.79 | 0.004 |
| M |  |  | <0.0001 |  |  | <0.0001 |
| M0 | Reference |  |  | Reference |  |  |
| M1 | 15.87 | 12.11-20.80 | <0.0001 | 3.60 | 2.05-6.32 | <0.0001 |
| Subtype |  |  | <0.0001 |  |  | <0.0001 |
| HR+/HER2- | Reference |  |  | Reference |  |  |
| HR+/HER2+ | 2.09 | 1.47-2.96 | <0.0001 | 1.31 | 0.89-1.94 | <0.0001 |
| HR-/HER2+ | 2.77 | 1.023-7.49 | 0.044 | 0.91 | 0.28-2.96 | 0.171 |
| HR-/HER2- | 12.15 | 7.72-19.13 | <0.0001 | 9.10 | 5.07-16.34 | 0.870 |
| Surgery |  |  | <0.0001 |  |  | <0.0001 |
| Yes | Reference |  |  | Reference |  |  |
| No/Unknown | 10.69 | 8.07-14.17 | <0.0001 | 2.37 | 1.55-3.63 | <0.0001 |
| Radiotherapy |  |  | 0.121 |  |  |  |
| Yes | Reference |  |  |  |  |  |
| No/Unknown | 1.25 | 0.94-1.66 | 0.122 |  |  |  |
| Chemotherapy |  |  | 0.007 |  |  | 0.009 |
| Yes | Reference |  |  | Reference |  |  |
| No/Unknown | 1.44 | 1.10-1.87 | 0.007 | 1.51 | 1.11-2.05 | 0.009 |
| Visceral metastasis |  |  | <0.0001 |  |  | 0.973 |
| No | Reference |  |  | Reference |  |  |
| Yes | 13.35 | 9.71-18.34 | <0.0001 | 0.96 | 0.35-2.59 | 0.928 |
| Unknown | 1.83 | 0.59-5.75 | 0.298 | 1.38 | 0.06-30.27 | 0.839 |
| Bone |  |  | <0.0001 |  |  | 0.057 |
| No | Reference |  |  | Reference |  |  |
| Yes | 15.63 | 11.71-20.86 | <0.0001 | 1.62 | 0.96-2.72 | 0.068 |
| Unknown | 1.37 | 0.34-5.54 | 0.658 | 0.20 | 0.02-2.12 | 0.180 |
| Liver |  |  | <0.0001 |  |  | 0.143 |
| No | Reference |  |  | Reference |  |  |
| Yes | 17.76 | 10.28-30.69 | <0.0001 | 1.91 | 0.81-4.52 | 0.198 |
| Unknown | 1.96 | 0.73-5.28 | 0.183 | 1.16 | 0.79-4.08 | 0.111 |
| Lung |  |  | <0.0001 |  |  | 0.554 |
| No | Reference |  |  | Reference |  |  |
| Yes | 11.44 | 8.10-16.15 | <0.0001 | 0.78 | 0.32-1.90 | 0.590 |
| Unknown | 3.14 | 1.39-7.08 | 0.006 | 0.48 | 0.11-2.04 | 0.321 |
| Brain |  |  | <0.0001 |  |  |  |
| No | Reference |  |  | Reference |  | 0.050 |
| Yes | 28.21 | 14.71-54.09 | <0.0001 | 2.48 | 1.09-5.62 | 0.029 |
| Unknown | 1.93 | 0.72-5.20 | 0.193 | 4.03 | 0.48-33.69 | 0.198 |

BCSS=breast cancer-specific survival; MBC=male breast cancer; FBC=female breast cancer; DC=ductal carcinoma; LC=lobular carcinoma; ER=estrogen receptor; PgR=progesterone receptor; HER2=human epidermal growth factor receptor

**Supplementary Figure1. BCSS of MBC and FBC associated with molecular subtypes.**

(A) HR+/HER2- subtype. (B) HR+/HER2+ subtype. (C) HR-/HER2+ subtype. (B) HR-/HER2- subtype.


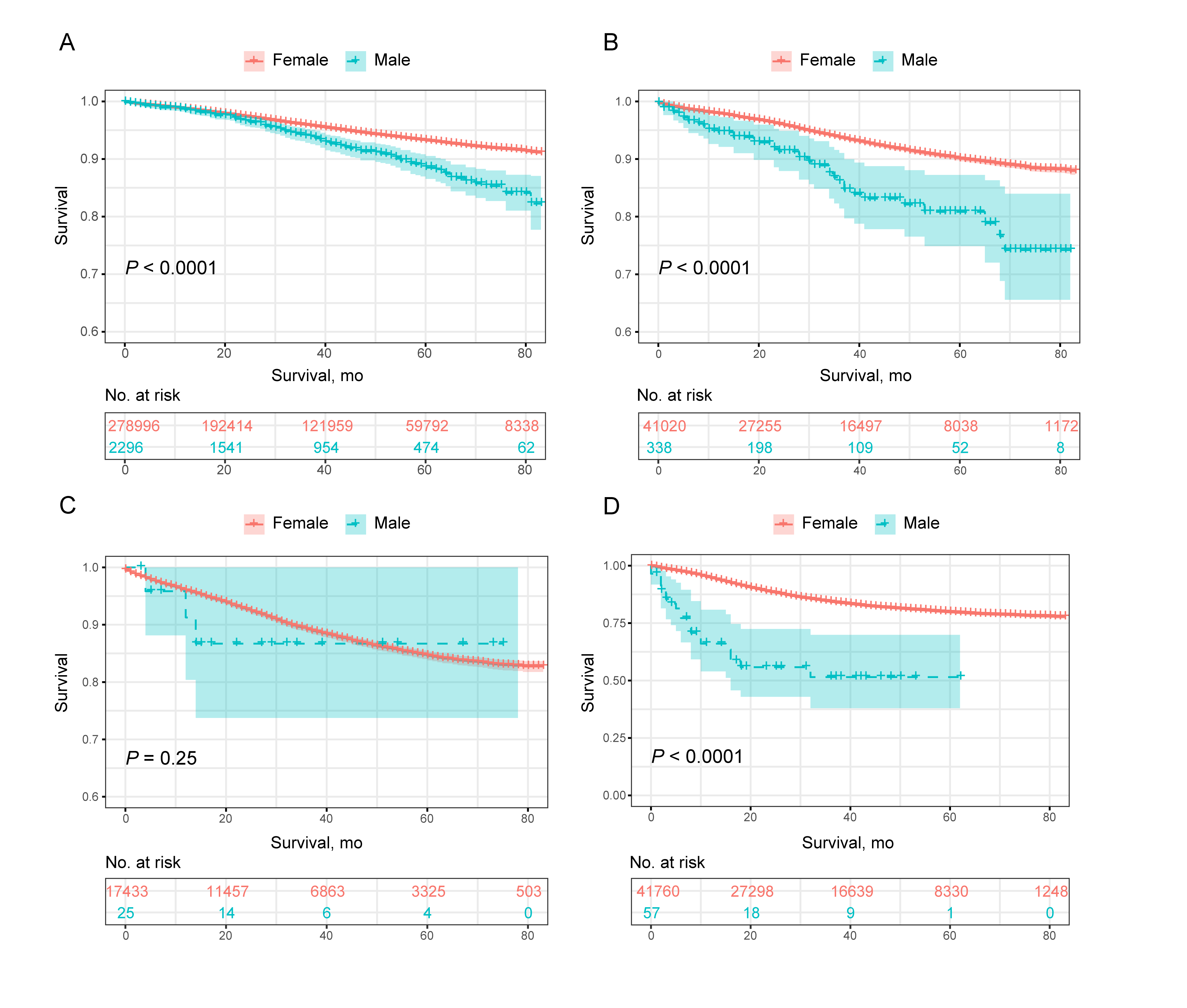
BCSS=breast cancer-specific survival; MBC=male breast cancer; FBC=female breast cancer.

**Supplementary Figure2. Overall prognosis of MBC and FBC regarding age.**

(A) OS in young cohort. (B) BCSS in young cohort. (C) OS in elderly cohort. (B) BCSS in elderly cohort.

OS=overall survival; BCSS=breast cancer-specific survival; MBC=male breast cancer; FBC=female breast cancer.

**
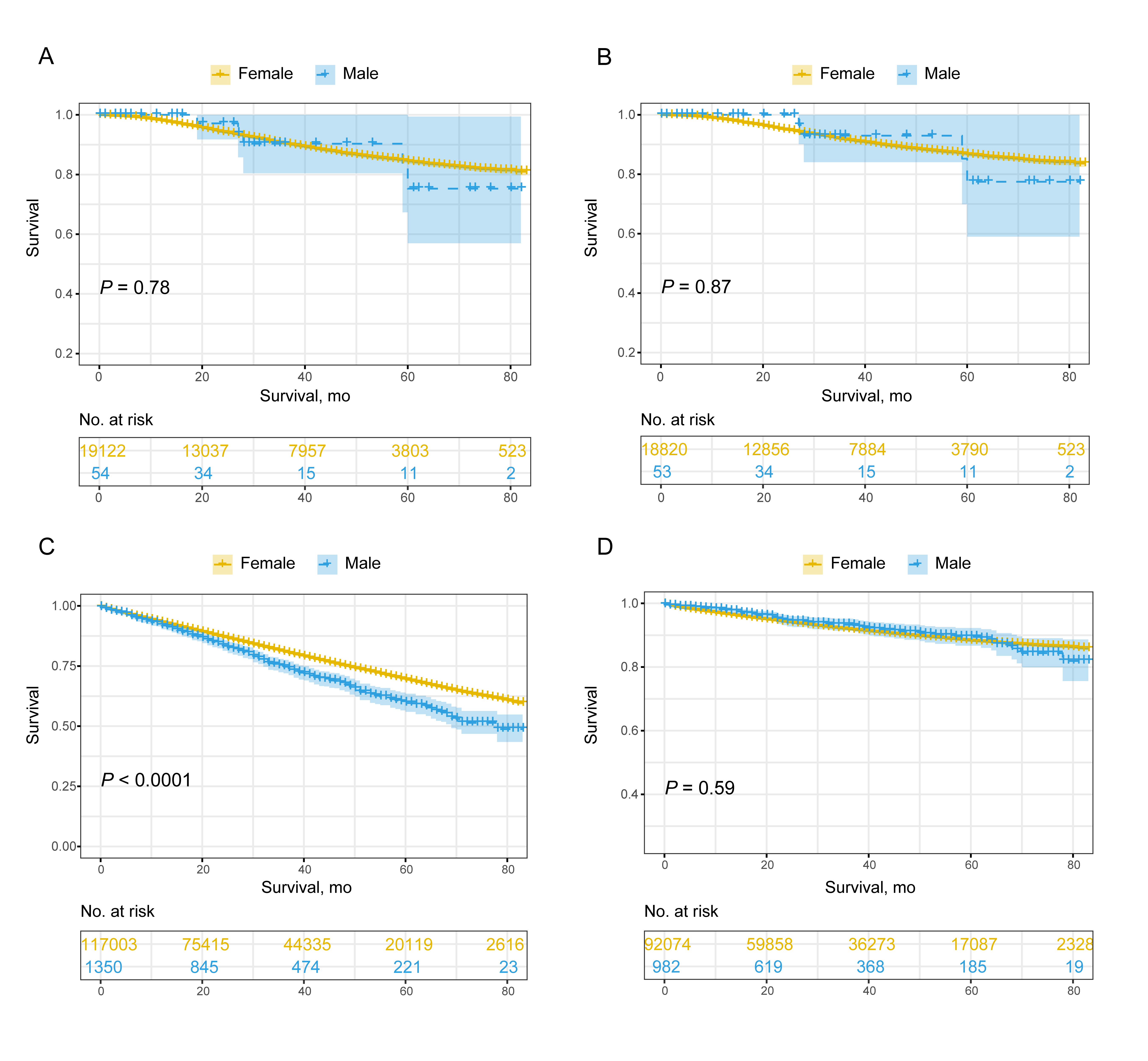
**

**Supplementary Figure3. Overall prognosis of MBC and FBC regarding therapeutics.**

**
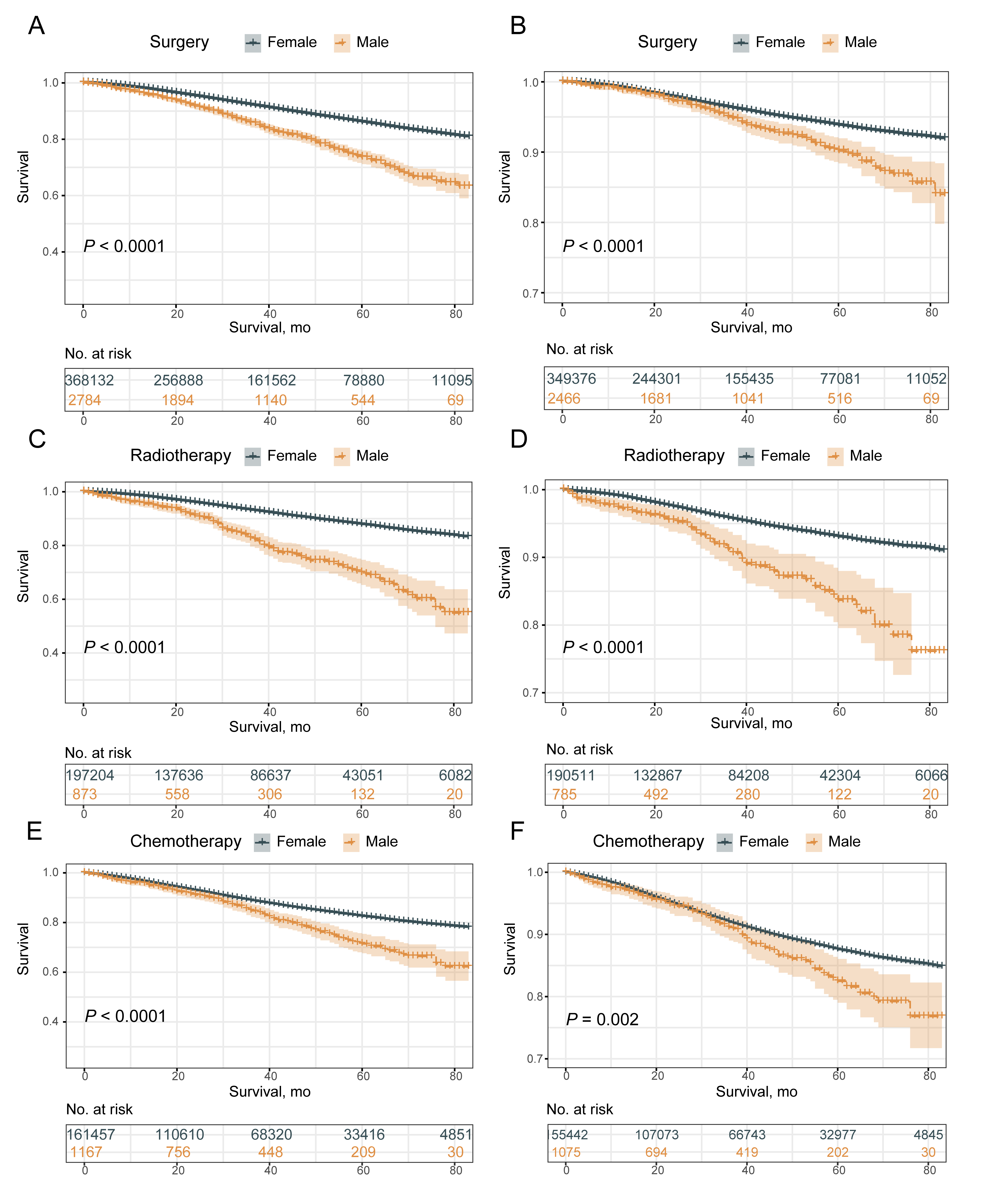
**(A) OS regarding surgery. (B) BCSS regarding surgery. (C) OS regarding radiotherapy. (D) BCSS regarding radiotherapy. (E) OS regarding chemotherapy. (F) BCSS regarding chemotherapy. OS=overall survival; BCSS=breast cancer-specific survival; MBC=male breast cancer; FBC=female breast cancer.

**Supplementary Figure4. OS of MBC and FBC regarding organ-specific involvement.**

(A) Visceral metastasis. (B) Bone-only disease. (C) Bone involvement. (D) Liver involvement. (E) Lung involvement. (F) Brain involvement.

OS=overall survival; MBC=male breast cancer; FBC=female breast cance
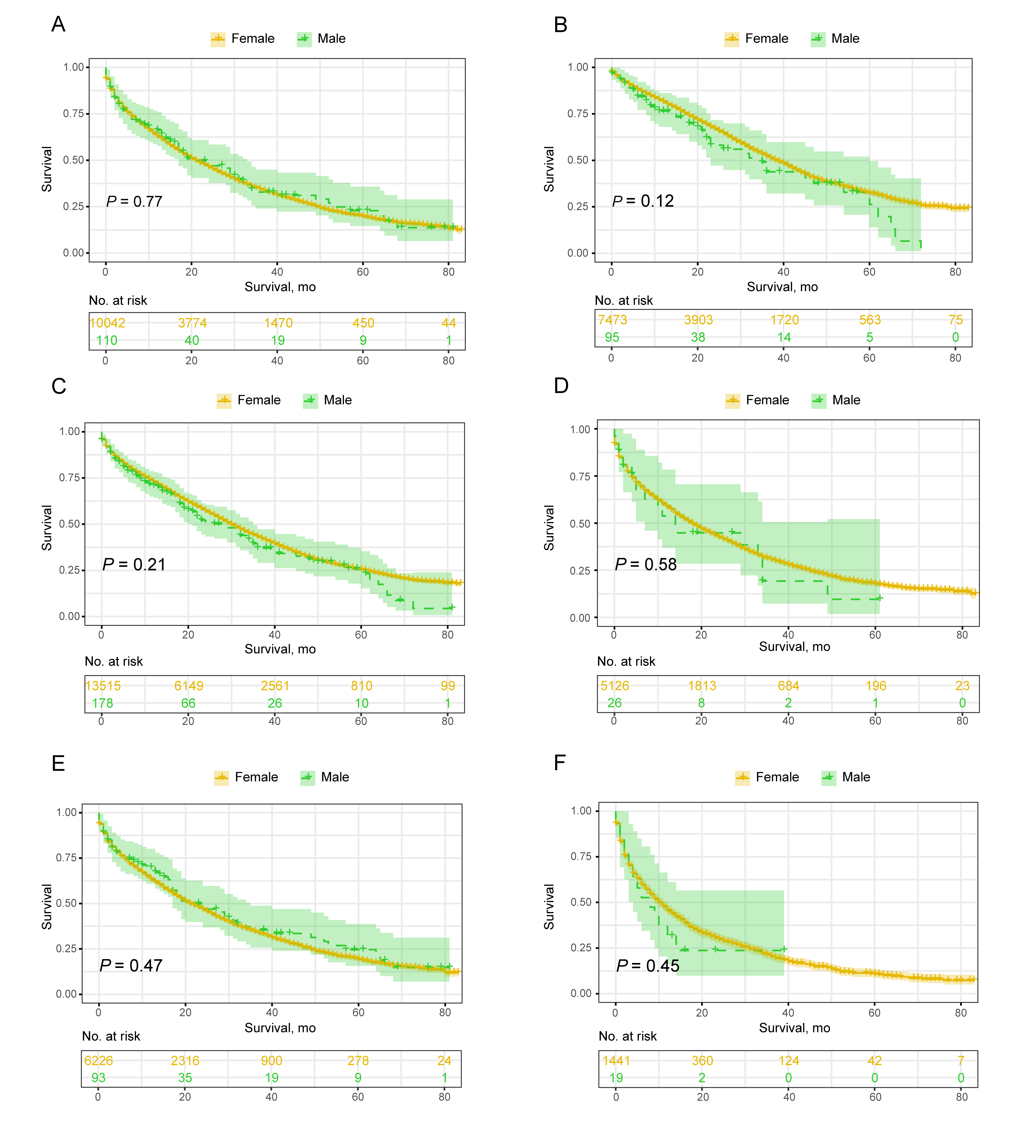
r.

**Supplementary Figure5. Overall prognosis of MBC and FBC associated with molecular subtypes after a 1:2 PSM.**

(A) OS. (B) BCSS. OS=overall survival; BCSS=breast cancer-specific survival; MBC=male breast cancer; FBC=female breast cancer; PSM=propensity score matching.

**
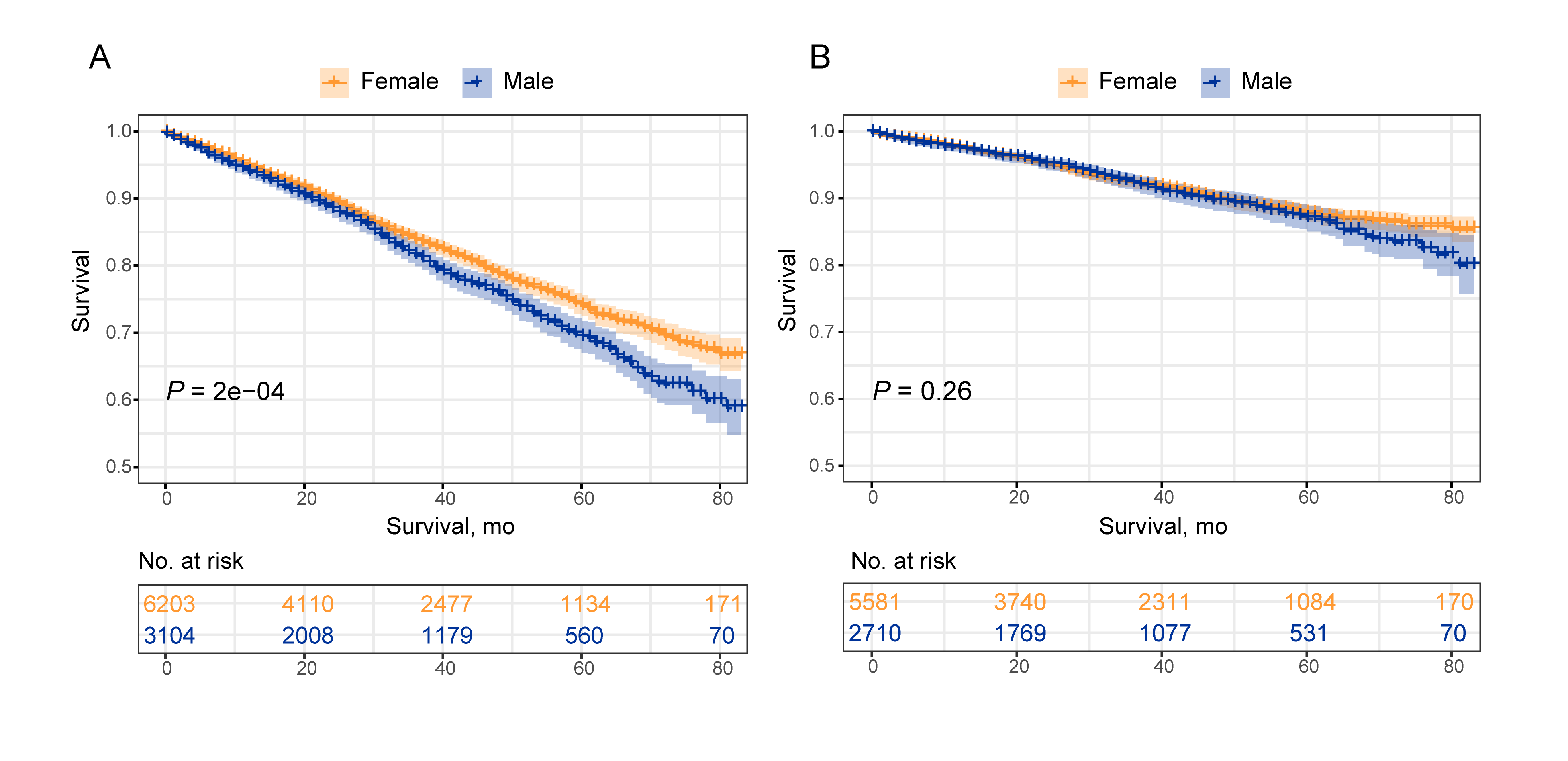
**

**Supplementary Figure6. OS of MBC and FBC associated with molecular subtypes after a 1:2 PSM.**

(A) HR+/HER2- subtype. (B) HR+/HER2+ subtype. (C) HR-/HER2+ subtype. (B) HR-/HER2- subtype.

OS=overall survival; MBC=male breast cancer; FBC=female breast cance**
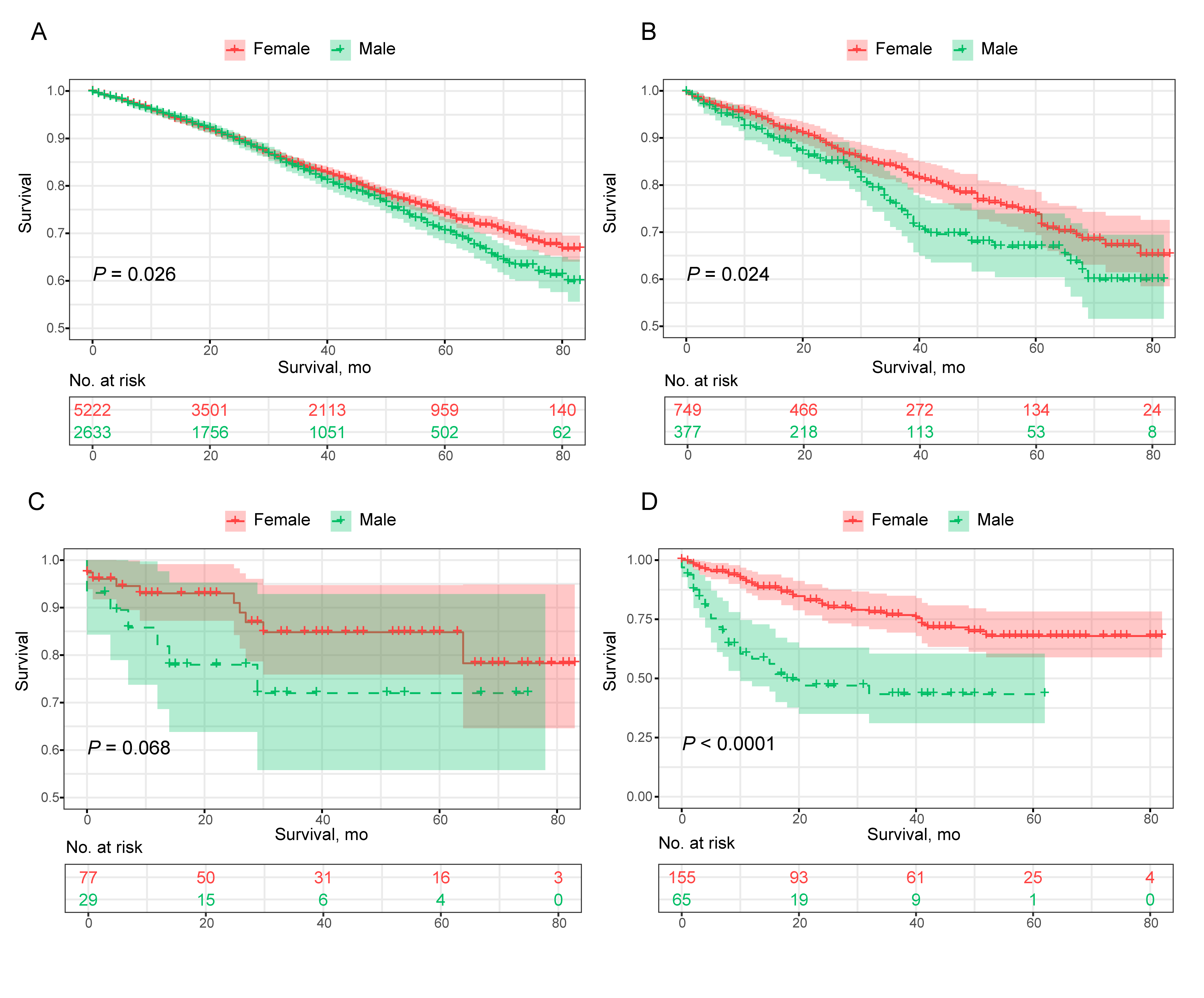
**r; PSM=propensity score matching; PSM=propensity score matching.

**Supplementary Figure7. BCSS of MBC and FBC associated with molecular subtypes after a 1:2 PSM.**

(A) HR+/HER2- subtype. (B) HR+/HER2+ subtype. (C) HR-/HER2+ subtype. (B) HR-/HER2- subtype.

BCSS=breast cancer-specific survival; MBC=male breast cancer; FBC=female breast cancer; PSM=propensity score matching. **
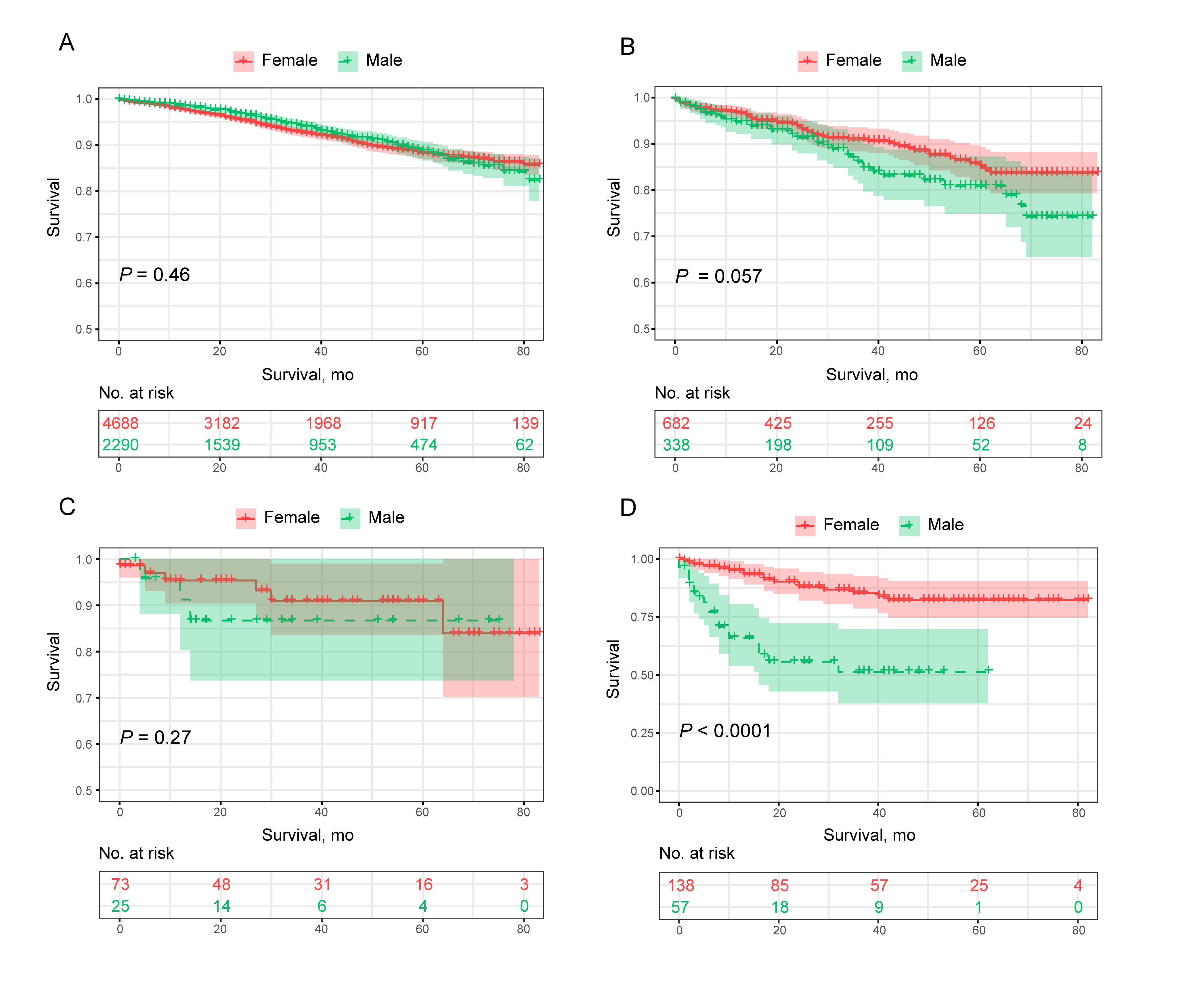
**

**Supplementary Figure8. Overall prognosis of MBC and FBC regarding age after a 1:2 PSM.**

(A) OS in young cohort. (B) BCSS in young cohort. (C) OS in elderly cohort. (B) BCSS in elderly cohort.

**
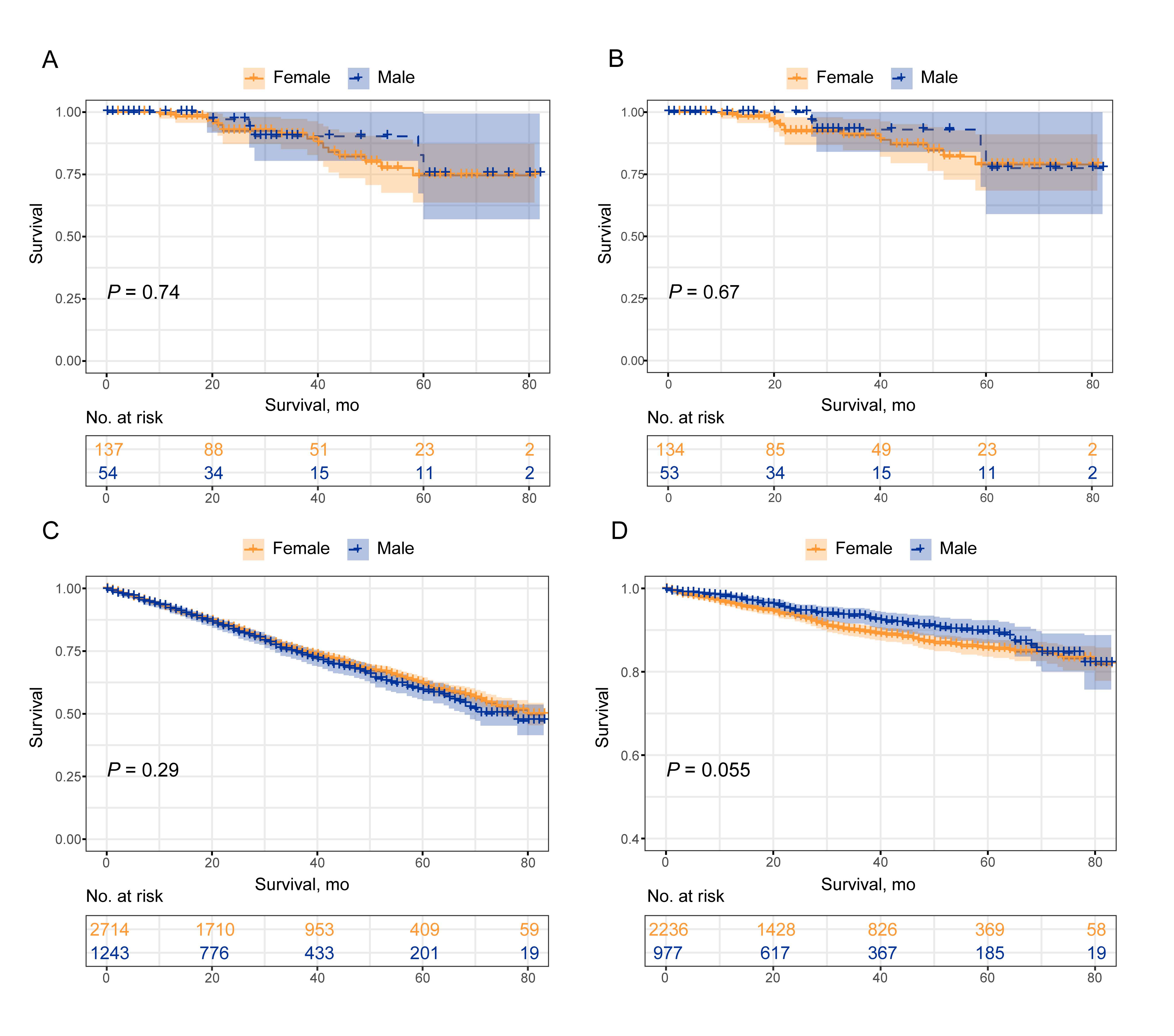
**OS=overall survival; BCSS=breast cancer-specific survival; MBC=male breast cancer; FBC=female breast cancer; PSM=propensity score matching.

**Supplementary Figure9. Overall prognosis of MBC and FBC regarding therapeutics after a 1:2 PSM.**

**
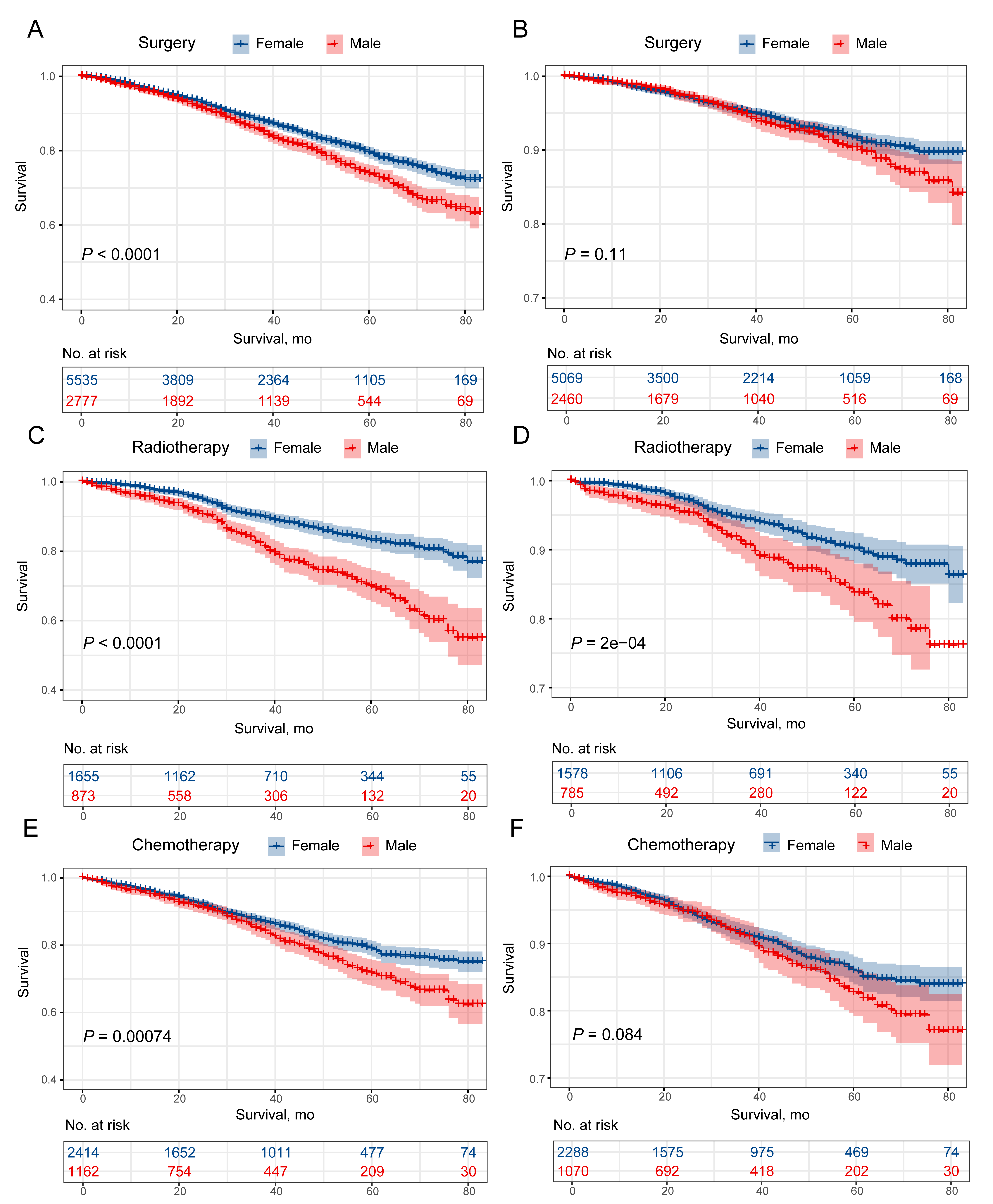
**(A) OS regarding surgery. (B) BCSS regarding surgery. (C) OS regarding radiotherapy. (D) BCSS regarding radiotherapy. (E) OS regarding chemotherapy. (F) BCSS regarding chemotherapy. OS=overall survival; BCSS=breast cancer-specific survival; MBC=male breast cancer; FBC=female breast cancer.
